# Quaternary ammonium salts based on caprylic acid as antimicrobial and surface-active agents†

**DOI:** 10.1039/d3ra07127h

**Published:** 2023-11-29

**Authors:** Marta Wojcieszak, Anna Syguda, Maciej Karolak, Łukasz Pałkowski, Katarzyna Materna

**Affiliations:** a Poznan University of Technology, Faculty of Chemical Technology Berdychowo 4 60-965 Poznan Poland katarzyna.materna@put.poznan.pl; b Department of Pharmaceutical Technology, Faculty of Pharmacy, Nicolaus Copernicus University Jurasza 2 85-089 Bydgoszcz Poland

## Abstract

In this work, amidequats and esterquats based on caprylic acid were investigated as promising compounds with surface properties and biological activity that are in harmony with the principles of green chemistry. Herein, caprylic acid, which is an essential component of the above compounds, is a noteworthy natural resource. Structural analysis was performed with the amphiphilic cations of the tested amidequats and esterquats, revealing two distinct factors, *i.e.*, the elongation of the alkyl chain and the presence of two different functional groups; these factors undoubtedly affect the desired biological activity. These compounds were synthesized and characterized in terms of their physicochemical properties, among which surface activity is pivotal. In addition, the surfaces of the tested compounds were investigated through a detailed topographical analysis. The obtained results suggested that the esterquats exhibited higher surface activity, wettability and foamability than the amidequats. Antimicrobial studies, on the other hand, are not as conclusive. For shorter chains, esterquats are more active than amidequats, while for longer chains (over C12), the trend was the opposite. The amidequats and esterquats presented in this research may be a potential good replacement for antimicrobial formulations or as alternatives to surface-active agents used in industry.

## Abstract 2

In this work, amidequats and esterquats based on caprylic acid were investigated as promising compounds with surface properties and biological activity that are in harmony with the principles of green chemistry.

## Graphical abstract

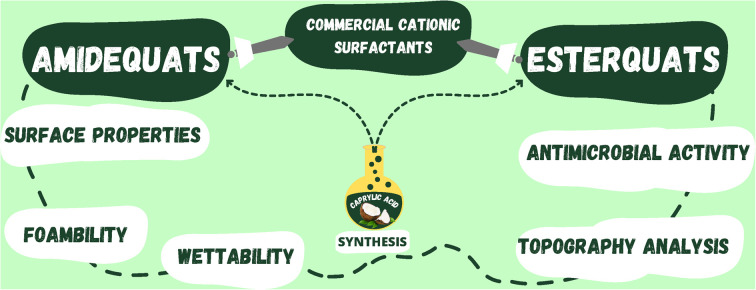

## 1. Introduction

Surfactants are common chemical compounds in many daily products.^1–3^ They are an inherent ingredient in detergents, cosmetics, household cleaners, pesticides, food, and drugs, among other products, and can be found in the textile, paper, or petrochemical industries.^4–7^ This wide spectrum of applications results from a number of properties that, in turn, are a consequence of the amphiphilic structure of surfactants. Due to their hydrophilic and hydrophobic parts, surfactants can reduce surface and interfacial tension, can wet a variety of surfaces, and tend to aggregate or adsorb.^3,7,8^ Currently, surface-active ionic liquids (SAILs) or surface-active quaternary ammonium salts (surface-active QASs), which exhibit surfactant-like behavior but are not conventional surfactants, are attracting much interest^9–11^ due to their amphiphilic structure. Notably, the physicochemical properties of this kind of compound can be strictly managed by the nature of the cations or anions.^11^ The literature is increasingly based on cations of natural origin, such as nicotine,^12^l-phenylalanine,^13^ glycine-betaine^14^ or anions, such as lauroyl sarcosinate.^15^ Para *et al.*^16^ described that the presence of labile moieties, acetal, amide, amine, ester, disulfide, ether or thioether moieties, in surface-active compounds could have a crucial impact on their surface properties as well as their physicochemical and biological functionalities. Recently, the most popular and commercially available compounds belong to the family of cationic esterquats.^17–20^ Their name is based on ester bonds located between quaternary ammonium groups and hydrocarbon tails. The second attractive and prospective structure is amidequats with an amide functional group. These compounds exhibit good biological activity against Gram-positive and Gram-negative bacteria. Due to their microbial effects, amidequats and esterquats display potential use as disinfectants.^21^

A noteworthy approach is focusing on industry's use of naturally derived compounds. This corresponds with the basic requirements of sustainable development and green chemistry necessary to reduce the environmental impact of the economy. The strategy outlined is evident in the global pharmaceutical and health care industry network. Caprylic acid is a natural compound with antimicrobial and anti-inflammatory properties.^22–25^ The first scientific reports on the antimicrobial properties of caprylic acid appeared in the 1940s and 1950s.^26,27^ This acid can be extracted from the milk of some mammals, as well as in coconut and palm oil.^28^ Akula *et al.* described that caprylic acid exhibits potential anticandidal activity against yeast. However, they stated that further microbiological and clinical evaluation are needed, which are essential for therapeutic application.^25^ The bactericidal effect of caprylic acid was also observed against *Escherichia coli*, *Salmonella enterica*, *Staphylococcus aureus* and *Listeria monocytogenes*.^29^ Moreover, caprylic acid is minimally soluble in water and exhibits a slightly unpleasant rancid-like smell and taste. Thus, there is a need to synthesize novel caprylic acid–based compounds with potential antibacterial and antifungal potential that can be exploited. Additionally, concerning the growing problem involving the resistance of organisms to treatment, the search for cost-effective, efficient chemical compounds with antimicrobial activity is justified.

The aim of this study was to evaluate a prospective group of esterquats and amidequats based on caprylic acid. A series of amidequats and esterquats were synthesized to study the effect of an additional functional group and alkyl chain elongation, which have a significant impact on micellization and analogous adsorption at the air/water interface; these properties determine the foaming ability and antimicrobial activity. The evaluation should guide the selection of future multifunctional compounds, *i.e.*, those that could potentially replace conventional compounds used as disinfectants, foaming agents, and surfactants.

At present, some researchers are interested in developing esterquats as prospective sustainability chemicals, but only a limited number of works have focused on amidequats. Therefore, in the present study, we widely analyze both groups of compounds and attempted to fill this gap.

## 2. Experimental

### 2.1. Chemicals

#### 2.1.1 Materials

(2-Dimethylamino)ethylamine (CAS 108-00-9) 98%, (2-dimethylamino)ethanol (CAS 108-01-0) 99%, octanoyl chloride (CAS 111-64-8) 99%, 1-bromooctane (CAS 111-83-1) 99%, 1-bromononane (CAS 693-58-3) 99%, 1-bromodecane (CAS 112-29-8) 98%, 1-bromoundecane (CAS 693-67-4) 99%,1-bromododecane (CAS 143-15-7) 97%, 1-bromotetradecane (CAS 112-71-0) 97%, 1-bromohexadecane (CAS 112-82-3) 98%, benzalkonium chloride 95% (CAS 63449-41-2), didecyldimethylammonium chloride 50% (solution in isopropanol and water, 2 : 3) (CAS 7173-51-5), reagents for two-phase system titration: [dimidium bromide (CAS 95-518-67-2) 95%, patent blue V sodium salt (CAS 20262-76-4) 97%, sodium dodecylsulfate(vi) (CAS 151-21-3) 98%] were purchased from Merck (European market, Poznan, Poland). Sodium bicarbonate (CAS 144-55-8) 99% and solvents: acetone (CAS 67-64-1) 99%, ethyl acetate (CAS 141-78-6) 99%, chloroform (CAS 67-66-3) 98.5% were purchased from Avantor (Gliwice, Poland).

#### 2.1.2 Synthesis

##### 2.1.2.1 Synthesis of aminoamide and aminoester

In a 250 mL round bottom flask, 15 g of the appropriate amine [(2-dimethylaminoethyl)amine and (2-dimethylamino)ethanol] was added in 20 mL of chloroform and sodium bicarbonate in a 5% molar excess over the amine. The mixture was cooled in an ice-water bath to 0 °C. Octanoyl chloride in 20 mL of chloroform was then added dropwise with vigorous stirring in the stoichiometric amount of the amine. The reaction was carried out so that the temperature did not exceed 15 °C. After the entire volume of octanoyl chloride was added dropwise, the mixture was refluxed for 24 h. The precipitate was then filtered off, and the filtrate's solvent was evaporated by a rotary evaporator. Finally, the products were purified by vacuum distillation.

##### 2.1.2.2 Synthesis of amidequats and esterquats

Four grams of the aminoamide or the aminoester obtained in the previous step, 10 mL of acetone and a stoichiometric amount of 1-bromoalkane were placed in a 100 mL round bottom flask. Reactions were carried out for 7 days at room temperature. The progress of the quaternization reaction was monitored by TLC chromatography. A small amount of the reaction mixture to be tested was applied to a Merck silica gel 60 plate using a very thin capillary. The developing phase was the chloroform-methanol system in a volume ratio of 9 : 1. The dried plates were developed with iodine vapor. The basis for identifying the component was the distance the spot traveled from the starting point. Initial amines were used as an internal standard in the conducted studies. After 7 days, acetone was completely evaporated from the postreaction mixture using a rotary evaporator, and the raw products were placed in the refrigerator for 24 hours. Then, 10 mL of cold ethyl acetate was added to the flasks with the products. Next, the products were vacuum filtered and washed with cold ethyl acetate. Finally, the amidequats and esterquats were dried at 60 °C in a vacuum oven.

##### 2.1.2.3 Determination of cationic surfactants

The principle of the method consists in titration in a water-chloroform system of the quaternary ammonium salt solution with a standard solution of sodium dodecyl sulphate(vi) in the presence of a mixed indicator (cationic – dimidium bromide and anionic – patent blue V sodium salt). The cationic active substance forms a salt with the anionic indicator, which dissolves in chloroform, giving this layer a turquoise color. During the titration, the anion present in the titrant displaces the anion indicator from the salt and the turquoise color disappears from the chloroform layer as the dye passes into the aqueous phase. The excess of the titration solution forms a salt with the cationic indicator, which dissolves in the chloroform layer, dyeing it purple. 0.2 g (±0.0001 g) of the tested compound was placed in a 50 mL volumetric flask, a few drops of isopropanol were added, and then topped up with deionized water. Then, a 10 mL sample was taken from the prepared solution and placed in a conical flask with a ground stopper. In the next step, 15 mL of chloroform and 5 mL of the mixed indicator working solution were added to the Erlenmeyer flask. The obtained two-phase system was titrated with a 0.004 mol L^−1^ solution of sodium dodecyl sulphate(vi) until the color of the chloroform layer changed from turquoise to purple. During the titration, after adding subsequent portions of the titrant, the titrated solution was plugged with a stopper and shaken vigorously. The percentage of cationic surfactant content was calculated using the formula:
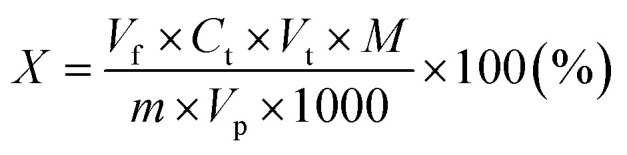
*V*_f_ – the volume of the flask with the solution of the substance being determined (mL), *C*_t_ – the concentration of the titrant (mol L^−1^), *V*_t_ – the volume of the titrant solution (mL), *M* – the molar mass of the analyzed compound (g mol^−1^), *m* – the mass of the tested sample (g), *V*_p_ – the volume of solution taken with the pipette (mL).

##### 2.1.2.4 Determination of water content

The water content was determined coulometrically using Karl Fischer titration (Metrohm type 702 SM Titrino, Switzerland). Coulometry is a method of electrochemical analysis in which the mass of a substance is determined on the basis of the measured electric charge needed to change the oxidation state of the substance being determined. A sample of the test compound in the form of a methanol solution was injected into the tank containing the titrant. Then, the sample mass was entered on the automatic titrator. During this time, the following reactions occurred:CH_3_OH + SO_2_ + C_5_H_5_N → [C_5_H_5_NH]^+^[CH_3_OSO_2_]^−^[C_5_H_5_NH]^+^[CH_3_OSO_2_]^−^ + H_2_O + I_2_ + 2C_5_H_5_N → [C_5_H_5_NH]^+^[CH_3_OSO_3_]^−^ + 2([C_5_H_5_NH]^+^I^−^)

The device automatically calculated the amount of water in ppm based on the sample weight entered, measuring the charge used to generate the titrant, which then reacts with the analyte. The water content in pure methanol was initially determined, and then in the prepared methanol solutions. Based on the collected results, the water content in the tested compounds was calculated.

##### 2.1.2.5 Determination of melting points

Melting points were determined with a Büchi Melting Point B-545 apparatus and are uncorrected.

##### 2.1.2.6 NMR spectroscopy

NMR spectra were recorded at 25 °C in a BRUKER ASCEND™ 400 MHZ NANOBAY spectrometer operating at 400 MHz and 100 MHz for ^1^H and ^13^C nuclei, respectively. Samples were dissolved in CDCl_3_ using tetramethylsilane (TMS) as an internal reference (*δ* = 0.00 ppm).

### 2.2. Surface activity studies

Surface activity measurements were performed mainly with a DSA 100 analyzer (Krüss, Germany, accuracy ± 0.01 mN m^−1^), in a Fisher brand FBH604 thermostatic bath (Fisher, Germany, accuracy ± 0.1 °C) to control the temperature. Surface tension was determined by the shape drop method. An image of the drop was created and digitized. The surface tension (*γ* in mN m^−1^) is determined by studying the profile of the drop based on the mathematical Laplace equation. Values of critical micelle concentration (CMC) and surface tension at the CMC (*γ*_CMC_) were designated by a linear regression analysis method. Crucial information about the Gibbs free energy of adsorption (Δ*G*^0^_ads_) and the adsorption efficiency, pC_20_, were described in our previous works.^30,31^

The wettability is based on contact angle (CA) measurements. They are based on a study of a drop deposited on a hydrophobic surface (paraffin). After the drop shape and contact line are correctly identified, the droplet shape is fitted to a mathematical model (Young's–Laplace fit).

All measurements (surface tension and contact angle) were conducted at 25 °C.

### 2.3. Foamability

The foaming properties of the compounds were tested using the method described in the BN-86 6140-08/03 (1986) standard. Measurements were carried out for four concentrations (4 g L^−1^, 2 g L^−1^, 1 g L^−1^ and 0.5 g L^−1^) of the tested solutions of the amidequats, the esterquats and the reference substances – didecyldimethylammonium chloride and benzalkonium chloride. Before the solutions were prepared for didecyldimethylammonium chloride, the isopropanol was completely evaporated. The tests were carried out using the Ross–Miles apparatus at 37 °C (±0.1 °C). For this purpose, the thermostat connected to the device was activated, and the appropriate temperature was programmed. The SIMAX bottle with the test compound solution was placed in the thermostat chamber. Then, 50 mL of the solution was taken from the bottle and slowly poured over the wall of the apparatus to prevent the generation of foam. Then, 200 mL of the tested solution was measured and introduced into the measuring dropper. The dropper was placed on top of the apparatus cylinder and the tap of the dropper was turned on. The solution was poured into the apparatus so that the stream did not flow down the walls but hit the surface of the liquid directly. After 30 seconds, the height of the foam formed in the apparatus cylinder was measured. Then, after 5 minutes, the foam heights were measured again. Measurements were performed 4 times for each solution at each concentration. The average of four measurements was taken as the final result. Based on the data obtained, the following was calculated:

(a) Foaming ability (FA) from the formula:
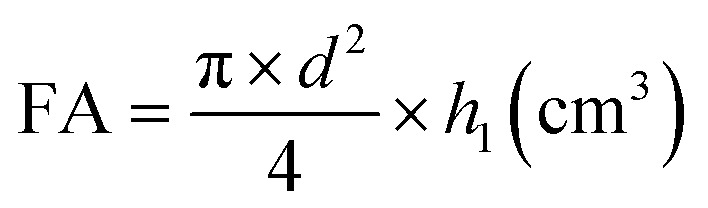
where: *d* – internal diameter of the cylinder of the Ross–Miles apparatus (cm), *h*_1_ – foam height read after 30 s (cm).

(b) Foam durability index (FDI) from the formula:
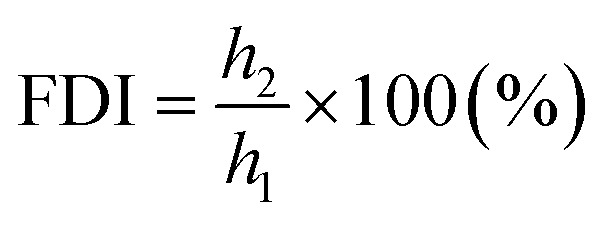
where: *h*_1_ – foam height read after 30 s (cm), *h*_2_ – foam height read after 5 min (cm).

### 2.4. Atomic force microscopy

The samples of studied compounds were dissolved in water and then small volume (25 μl) of studied solutions in different concentration of IL's were applied with a syringe to a piece (1 × 1 cm) of freshly cleaned mica, which had previously been glued to a steel disc using double-sided tape. Samples prepared in this way were left to dry in the air for 24 hours. During drying, the samples were protected from possible contamination from the air. The microscope used for the study is an NX10 manufactured by Park Systems. Images were taken using non-contact mode. The microbeams used in the measurements are All-In-One D manufactured by Budgetsensors. Their nominal elastic constant is 40 N m^−1^. The tests were performed at a room temperature of about 23 °C. The resolution of every picture was 512 × 512 px, while the scan speed was between 0.3 and 0.5 Hz. For each sample, 15 × 15, 5 × 5, 2 × 2 and 0.5 × 0.5 micrometer images were taken. The images were processed using Gwyddion software.

### 2.5. Antimicrobial activity studies

The antimicrobial activity of the analyzed compounds was tested against: *Staphylococcus aureus* ATCC 25213 (SAU), *Pseudomonas aeruginosa* ATCC 27853 (PAE), *Klebsiella pneumoniae* ATCC 700603 (KPN), *Escherichia coli* ATCC 25922 (ECO), *Enterococcus faecalis* ATCC 29212 (EFA), *Candida albicans* ATCC 90028 (CAL), and *Pseudomonas putida* ATCC 49128 (PPT). The strains were purchased from Microbiologics (San Diego, CA, USA). Bacterial and fungal suspensions with a turbidity equivalent to that of a 0.5 McFarland standard (approx. cell density 1.5 × 10^8^ CFU mL^−1^) were prepared by suspending growth from blood Trypticase Soy Agar (TSA, BBL) plates in 2 mL of sterile saline. The antibacterial effects of the analyzed compounds were evaluated by determining the minimal inhibitory concentration (MIC, % w/v) values, which ranged from 1.0 to 0.000125% w/v, using the disk-diffusion method and the control plate without disinfectant. The test medium was Mueller–Hinton agar (bio-Merieux). The MIC was determined as the lowest concentration of the tested agent at which no visible bacterial growth could be detected. A barely visible haze of growth was disregarded. MIC was read at 37 °C after 24 or 48 h of culture for the other bacteria and for *Candida* spp. Moreover, it should be noted that some compounds showed lower antimicrobial activity than that of the remaining tested substances for which antimicrobial activity was not tested at higher concentrations. Therefore, their activity is marked as ‘above a given value’. Details of the agar dilution procedure for testing microorganisms have been published by the Clinical and Laboratory Standards Institute.^32^

## 3. Results and discussion

### 3.1. Synthesis

**Fig. 1:**
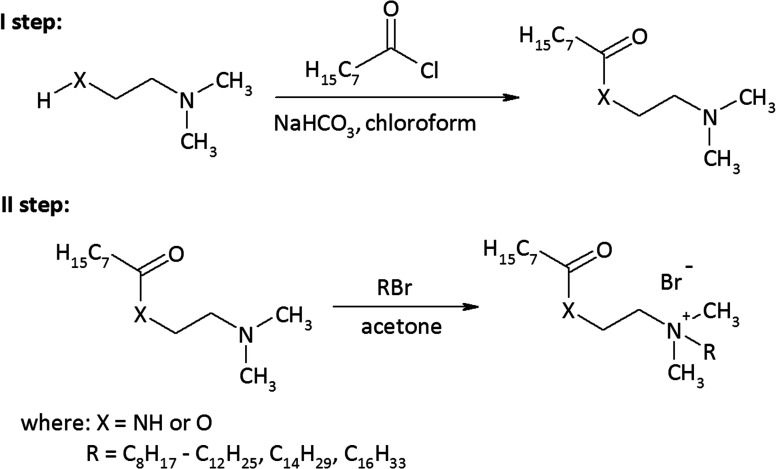
Synthesis of the amidequats and esterquats.

As a result of the reaction of (2-dimethylaminoethyl)amine and (2-dimethylamino)ethanol with the acid chloride of caprylic acid in the presence of sodium bicarbonate (Fig. 1), aminoamide and aminoester were obtained. To purify the aminoamide and the aminoester, the compounds were distilled under reduced pressure. The boiling point for *N*-[(2-dimethylamino)ethyl]octanamide was 156–159 °C at 5 hPa, while for (2-dimethylamino)ethyl octanate, it was 114–116 °C at also 5 hPa. The higher boiling point of the aminoamide in relation to the aminoester is the result of the formation of hydrogen bonds in the aminoamide molecule between the nitrogen atom of the N–H group and the oxygen atom of the carbonyl group, as is the case with carboxylic acids that form dimers. The resulting tertiary amines were colorless liquids. *N*-[(2-Dimethylamino)ethyl]octanamide was obtained with a yield of 96%, and (2-dimethylamino)ethyl octanate was obtained with a yield of 94%.

The second stage of the amidequats and the esterquats synthesis involved a quaternization reaction between the aminoamide and the aminoester with 1-bromoalkanes that contained 8 to 16 carbon atoms in the alkyl chain (Fig. 1). Four amidequats (AC10, AC12, AC14, AC16) and one esterquat (EC12) have been previously described.^21,33^ The other compounds are new. The yields of the obtained amidequats ranged from 90 to 94%, while for the esterquats, they were lower and amounted to 81–94% (Table 1). This difference in the efficiency of these two groups of compounds is related to their purification from the postreaction mixture with ethyl acetate, in which the esterquats dissolved more easily and losses occurred during their washing. A greater hygroscopicity of the esterquats in comparison to the amidequats was also noticeable. All obtained quaternary ammonium bromides were white solids, and the amidequats were characterized by melting points approximately 100 °C lower than those of their esterquat analogs (Table 1). In fact, it would be more expected that the additional H-bonding offered by the amide group would increase the melting point, but the opposite was observed. Differences in the melting points of the compounds could suggest two different conformations of these two groups of compounds. This is probably related to prototropic tautomerism, which can occur in amidequats and is not possible in the case of esterquats due to the lack of a hydrogen atom next to the oxygen atom. The tendency of the hydrogen atom to contact oxygen in amidequats causes the molecule to be positioned in such a way that the hydrogen atom is located as close as possible to the oxygen atom of the carbonyl group. It is then possible to migration the hydrogen atom from the N–H group to the oxygen of the carbonyl group and form an N=C double bond. Tautomers can transform into each other as a result of a spontaneous intramolecular reaction. The presence of a double bond in one of the tautomers between the carbon atom and the nitrogen atom results in the formation of a “pseudounsaturated” compound. Additionally, the double bond between the carbon atom and the nitrogen atom may generate the “*cis*” position relative to each other of functional groups containing long alkyl substituents. This probably has the effect of lowering the melting point of amidequats relative to their esterquat analogues. The phenomenon of prototropic tautomerism is not observed in esterquats. Due to the stronger van der Waals forces, these compounds therefore have higher melting points. Due to the fact that the melting points of amidequats are below 100 °C, they can be classified as ionic liquids. For all amidequats and esterquats, the cationic substance content was determined by two-phase titration. The results were in the range of 92–97%. The residue (supplement to 100%) is water, which was confirmed by ^1^H NMR and Karl Fischer titration.

**Table 1:** Melting point, surfactant content and reaction yield of the synthesized amidequats (AC8–AC16) and the esterquats (EC8–EC16) based on caprylic acid

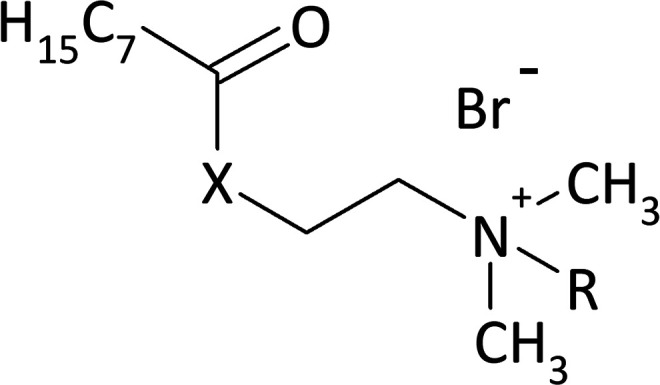				
Abbreviation	R	Melting point (°C)	Surfactant content (%)	Yield (%)
**X = N–H AMIDEQUATS**				
AC8	C_8_H_17_	49.0–49.1	95.0	90
AC9	C_9_H_19_	53.0–53.7	96.0	90
AC10	C_10_H_21_	52.5–53.5	96.5	91
AC11	C_11_H_23_	56.6–56.9	96.5	92
AC12	C_12_H_25_	54.8–55.2	97.0	93
AC14	C_14_H_29_	55.4–56.3	97.0	93
AC16	C_16_H_33_	59.0–59.7	96.5	94

**X = O ESTERQUATS**				
EC8	C_8_H_17_	149.3–153.1	92.0	81
EC9	C_9_H_19_	155.0–157.5	92.5	82
EC10	C_10_H_21_	156.2–158.9	94.0	84
EC11	C_11_H_23_	156.9–159.8	94.5	85
EC12	C_12_H_25_	158.1–160.4	96.5	90
EC14	C_14_H_29_	160.0–163.7	95.0	94
EC16	C_16_H_33_	161.2–164.1	97.0	94

### 3.2. Surface properties

**Fig. 2:**
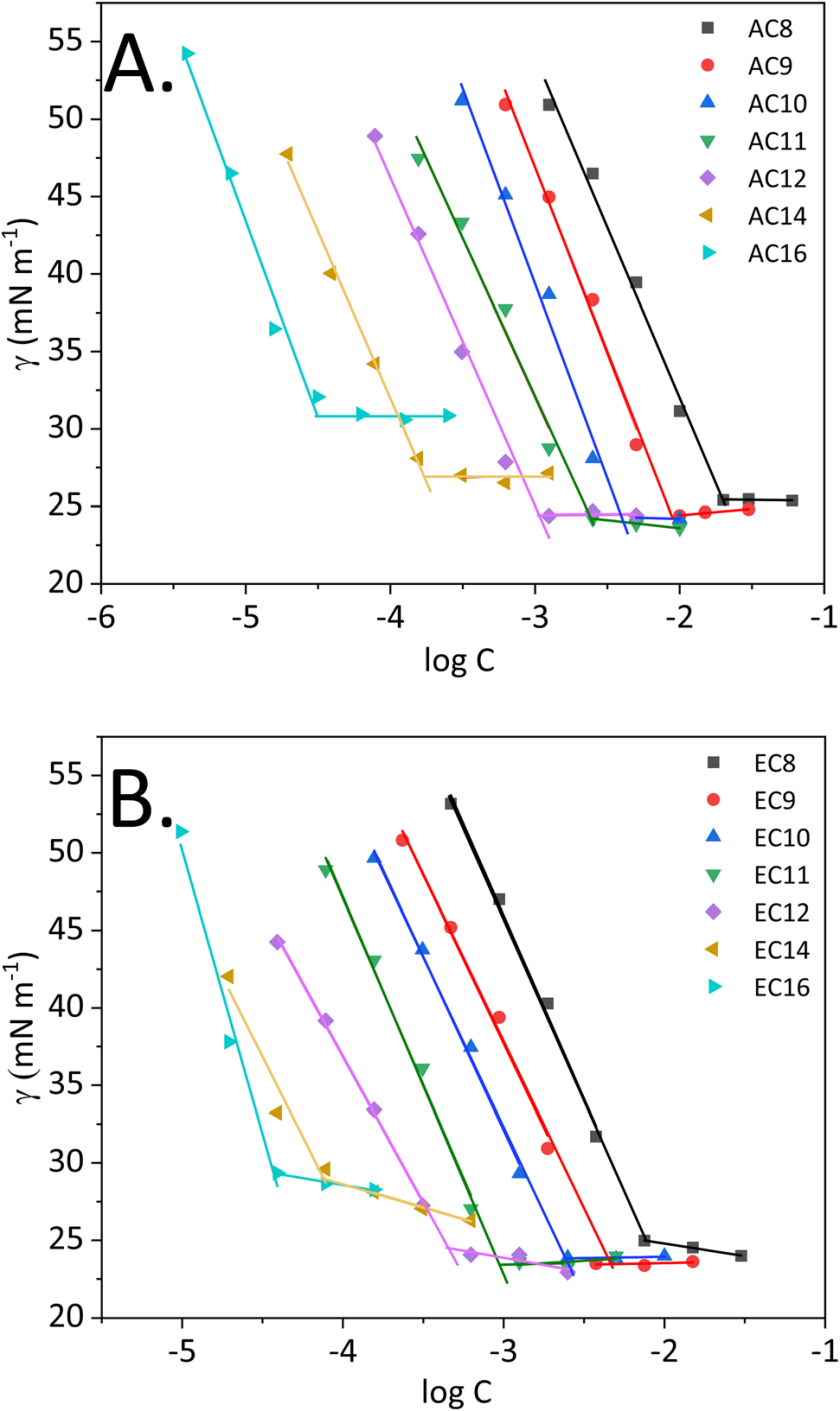
Surface tension *vs.* logarithm of concentration of the analyzed compounds ((A) SAILs and (B) QASs).

Surface tension measurements are useful for characterizing interfacial phenomena, including assessing adsorption at the boundary surface or associating amidequats and esterquats in a liquid environment of high polarity, such as water. From a theoretical point of view, the entire process depicting the schematic of micelle formation was described in our recent works.^30,31,34^Fig. 2 shows the surface tension (*γ*) as a function of logarithm of concentration (log *C*) for the studied compounds at 25 °C. In this work, it is worthwhile to explain the process, which occurs in aqueous solution, since it is crucial for compounds that show surface activity. During the process, which ends in micellization, several key stages can be distinguished. The first is associated with a marked decrease in surface tension, which can be observed for all synthesized compounds. Then, a transition to a virtually constant surface tension is observed. According to the theory of molecular self-organization reported in the literature,^8,35,36^ this behavior results from the formation of a hydrophilic–hydrophobic monolayer at the free air–IL interface in solution. The next step focused on saturating this interface. After it is saturated with the molecules of the compound, self-organized single molecules are formed in the analyzed solution first, followed by micelles. Moreover, for each compound, it is possible to determine the concentration at which micellization occurs, *i.e.*, the CMC, as discussed in detail later in this paper.

Nevertheless, it is important to realize that the amphiphilic structure and behavior of our compounds allow them to be classified as surface-active compounds. Taking into consideration the structure of the synthesized SAILs and QASs, in which the bromide anion remains unchanged, but the cation is modified, special attention will be focused on analyzing surface activity based on the cationic structure. However, some scientific reports^8,9,35^ indicate that for halides (which include bromides), the size and propensity for polarizability of the anions are key. Consequently, our bromide-based compounds that are larger and more polarizable can lead to stronger micellar interactions than that of chlorides, for example see ref. 9.

The values of the surface–activity parameters of the tested compounds are listed in Table 2.

**Table 2:** Surface properties of amidequats and esterquats.^a^ All values obtained from surface tension measurements

Abbreviation		CMC (mmol L^−1^)	pC_20_	*γ*_CMC_ (mN m^−1^)	CA (°)	Δ*G*^0^_ads_ (kJ mol^−1^)
**AMIDEQUATS**						
SAILs	AC8	20.00	2.93	25.4	29.71	−23.60
	AC9	8.51	3.24	24.4	28.57	−24.00
	AC10	3.98	3.52	24.3	24.88	−24.42
	AC11	2.41	4.00	24.2	26.44	−28.44
	AC12	0.88	4.25	24.5	24.48	−28.23
	AC14	0.17	4.96	26.8	33.96	−29.08
	AC16	0.03	5.36	30.9	57.10	−28.30

**ESTERQUATS**						
QASs	EC8	7.71	3.28	25.0	19.05	−22.99
	EC9	4.52	3.68	23.5	17.22	−26.07
	EC10	2.35	3.91	23.8	20.48	−28.94
	EC11	0.96	4.23	23.4	18.61	−28.76
	EC12	0.45	4.83	24.5	23.03	−30.71
	EC14	0.08	5.26	29.0	32.61	−31.31
	EC16	0.04	5.07	29.7	50.28	−29.33
	BAC	4.29	3.23	36.5	67.90	−27.03
	DDAC	1.69–2.00^b^	4.13	27.1	41.03	−32.31
	DomphB	1.78^c^		36.5^c^		
	C_10_TAB	67.00^d^		40.0^d^		
	C_12_TAB	15.00^d^		33.2–39.0^d^		
	C_14_TAB	4.08^e^		37.9–38.1^g^		
	C_16_TAB	0.97–1.00^f^		41.0^h^		

a BAC – benzalkonium chloride, DDAC – didecyldimethylammonium chloride, DomphB – domiphen bromide, C_10_TAB – decyltrimethylammonium bromide, C_12_TAB – dodecyltrimethylammonium bromide, C_14_TAB – trimethyltetradecylammonium bromide, C_16_TAB – hexadecyltrimethylammonium bromide. The accuracy of contact angle (CA) values ± 0.6°.

b From ref. 37.

c From ref. 38.

d From ref. 39.

e From ref. 40.

f From ref. 41.

g From ref. 42.

h From ref. 43.

The surface tension at CMC (*γ*_CMC_) of the analyzed SAILs and QASs decreased from the value of water (72.8 mN m^−1^) to a minimum ranging from 24.2 to 30.9 mN m^−1^ (SAILs) and from 23.4 to 29.7 mN m^−1^ (QASs), at which a plateau occurred. Notably, the difference between the lowest and the highest value of *γ*_CMC_ was almost 7 mN m^−1^. In this situation, the effect of higher *γ*_CMC_ values for the long-chain homologs may be related to the melting points of amidequats and esterquats. As described by Rosen,^8^ along with the elongation of the alkyl chain, the melting point of the compounds with surface activity should increase, leading to a tendency to form liquid crystal phases in solution. Admittedly, in our study, the *T*_m_ value is higher for EC14 and EC16 than the other esterquats, which exhibit a *T*_m_ at a similar level. In contrast, for AC16, the *T*_m_ value is the highest. For AC14, the trend is clearly unobservable. Perhaps this SAIL, despite the long alkyl chain in the cation, has less tendency to form liquid crystalline phases in solution; alternatively, the effect results from the formation of additional interactions, as suggested by the literature.^8^

The data presented in Table 2 clearly confirm that amidequats and esterquats exhibit excellent surface activity. It should be emphasized, however, that an important aspect is the structure of the tested compounds. Esterquats contain two oxygen atoms, which were likely to promote the formation of interactions between the compound molecules and the solvent. In other words, esterquats exhibited a better ability to micellize at relatively low concentrations. Undoubtedly, this phenomenon is reflected in the values of parameters describing surface activity. The above statement is based on the obtained CMC values.

The Klevens equation is as follows: log CMC = *A* − *Bn*, where *A* and *B* are parameters that are constant for a particular homologous series at a constant temperature. *A* is responsible for the contribution of the polar head to micelle formation, whereas *B* is related to the average contribution to micelle formation of each additional methylene group in the hydrophobic chain.^13,44^ Based on the empirical Klevens equation, the variation in the CMC depends on the hydrocarbon chain length.^45^ To check the above assumption, the Klevens dependency for the synthesized amidequats and esterquats was determined (see Fig. S.33 in ESI Data†).log CMC_amidequats_ = −0.3481*x* + 1.1055log CMC_esterquats_ = −0.3104*x* + 0.4032

Both series have a slope of less than −0.35, which is similar to ester- or amide-functionalized surface-active compounds (slope *ca.* −0.40).^13^ Moreover, conventional amphiphilic compounds form almost parallel plots (slope *ca.* −0.30).^13,40,44^ In contrast, for amidequats or esterquats, the slope may be steeper than parallel, as has been recorded for l-phenylalanine quaternary ammonium salts based on ester bonds, for example.^46^

**Fig. 3:**
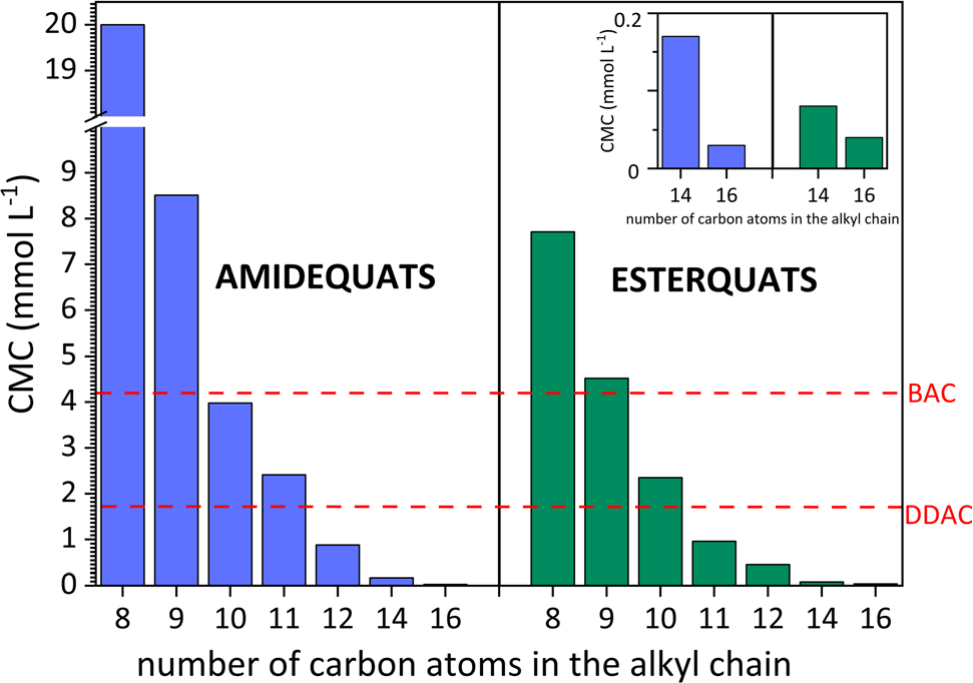
Critical micelle concentration (CMC) *vs.* the elongation of the alkyl chain of amidequats and esterquats and comparison of CMC values with other commercial surfactants.

For all compounds, the CMC values decrease with the elongation of the alkyl chain (see Fig. 3), which indicates that there is a strict correlation between CMC and the hydrophobic chain of surface-active compounds. In summary, the longer the alkyl chain is, the lower the CMC.^47^ The above discussion focused on the elongation of the alkyl chain and its effect on the micellization process. Nevertheless, in terms of structure, the studied compounds contain two substituents linked to both sides of the positively charged nitrogen. Based on Table 2, the highest values of CMC were noted in the situation when the lowest number of carbon atoms was incorporated in the structure of the amphiphilic cation (20.00 mmol L^−1^ for AC8). On the other hand, the lowest CMC values (0.03 mmol L^−1^ for AC16 and 0.04 mmol L^−1^ for EC16) were obtained for SAILs and QASs containing the highest number of carbon atoms in the substituents. Considering the ratio of the number of carbon atoms between the two substituents (amide- or ester-functionalized groups incorporated in one substituent and the second based on the alkyl chain), it is difficult to find a universal trend; however, for compounds in which the difference in carbon atoms between the substituents is 4 or more, the CMC value generally decreased dramatically for these SAILs and QASs.

According to Baltazar *et al.*^48^ and our suggestion, considering the total number of carbon atoms on either side of the positively charged nitrogen, it is clear that asymmetric substitution of long alkyl substituents leads to easier micellization with lower CMC values. On the other hand, the CMC values cannot be accurately estimated through the total number of carbon atoms in both substituents; compared to the CMC value results, the CMC values may demonstrate a more independent than cooperative effect.^48^

In addition, the surface activity of the tested amidequats and esterquats was compared with conventionally available cationic surfactants and widely used biocides, *i.e.*, didecyldimethylammonium chloride (DDAC) and benzalkonium chloride (BAC). Additionally, in this work, we compile cationic amphiphilic compounds, such as domiphen bromide (DomphB) and a homologous series of alkyltrimethylammonium bromides, namely, C_10_TAB, C_12_TAB, C_14_TAB, and C_16_TAB, which are commonly used in industry and have similar structures to the studied SAILs and QASs.

As shown in Fig. 3, some SAILs and surface-active QASs display superior surface properties to those of DDAC and BAC. This is certainly influenced by the amphiphilic structure of the synthesized compounds, especially the length of the hydrophobic chain. In detail, BAC contains only one long alkyl substituent, while the DDAC structure includes two substituents containing 10 carbon atoms. As a result, the CMC values of the analyzed SAILs and surface-active QASs were higher than BAC but only for EC8, EC9, AC8 and AC9. After comparing the CMC value of DDAC with the synthesized compounds, it was noticed that the CMC values were higher from AC8 to AC11 for amidequats and only from EC8 to EC10 for esterquats.

It is interesting to compare amidequats and esterquats with the series of quaternary ammonium bromides mentioned above. Additionally, the literature data^13,49^ described the evaluation between conventional surfactants with alkyl chains and compounds with ester or amide groups in the side chain. Due to the obtained results and based on research by Kapitanov *et al.*,^13^ ester or amide groups in the side chain impact polarity, which is relatively higher than that of alkyl chains. This is due to the intramolecular H-bonding between water and surface-active QASs (ester- or amidequat). As discussed above, the presence of amide or ester moieties has a significant impact on micellization and an analogous impact on adsorption at the air/water interface.

Another aspect worth considering is the presence of an aromatic ring as a substituent. In light of the extensive literature, compounds with aromatic cations or aromatic rings as substituents show higher surface activity than that of conventional quaternary ammonium surfactants. This results from the delocalized charge of the amphiphilic cation, which weakens the electrostatic repulsion between the charged main groups and promotes the aggregation process to occur.^9^ Admittedly, our esterquats and amidequats did not contain aromatic moieties. However, it is valuable to compare the surface activity of DomphB with EC12 and AC12 because these compounds contain one long alkyl substitute with the same number of carbon atoms. Despite the presence of a hydrophobic 2-phenoxyethyl group at the commercial surfactant headgroup, its tendency to adsorb at the air/water interface decreases. Most likely, this is because the bulky amphiphilic headgroup prevents water from interacting with the N^+^ atom. Consequently, the CMC values of DomphB were higher than those of EC12 and AC12, confirming the above conclusion.

Generally, the values of adsorption efficiency (pC_20_), as seen in Table 2, increased with increasing number of carbon atoms in the hydrophobic chain (EC16 is an exception). Moreover, Rosen^8^ suggested that the pC_20_ values often increase linearly with alkyl chain elongation, reflecting the negative free energy resulting from adsorption of a methylene group at these interfaces. For cationic amphiphiles, when the alkyl chain length increases by two additional carbon atoms, the pC_20_ values increase by 0.56–0.60 for adsorption in water solution–air. For the studied SAILs and QASs, the addition of two more carbon atoms to the alkyl chain appears to generally increase by no more than 0.76 (an exception was observed for EC10 and EC12, in which the difference between pC_20_ values was 0.92), indicating that the surface concentration, which is nearly the value of saturation, can be achieved with approximately 40% of the bulk phase of the compound concentration.^8^

The Δ*G*^0^_ads_ values are given in Table 2, and all the values for the amidequats and the esterquats were negative. This indicates that the adsorption phenomenon occurs spontaneously. Moreover, these Δ*G*^0^_ads_ values became more negative as the hydrocarbon chain increased.^45,50^

As is well known, the wettability of the tested surface is measured by the contact angle (CA). In Table 2, the values of CA ranged from 24.48 to 57.10° (the amidequats) and from 17.22 to 50.28° (the esterquats). These CA values are reserved for liquids that partially wet surfaces. Detailed research focusing on CA values is needed and will be discussed later in the work.

**Fig. 4:**
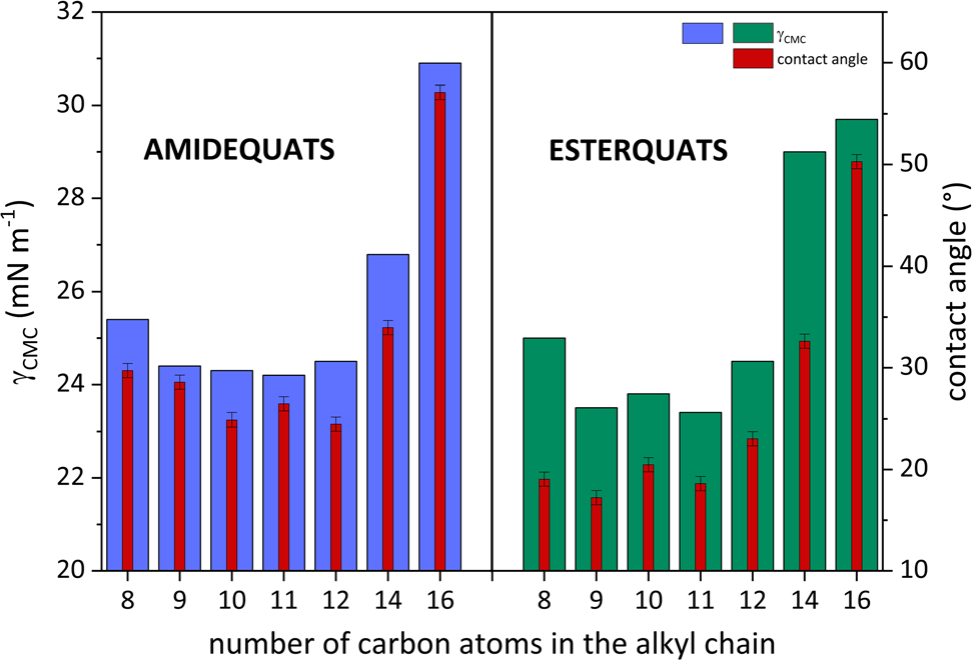
The left *Y*-axis shows the surface tension at CMC (*γ*_CMC_) as a function of the number of carbon atoms in the hydrophobic part of the structure. The right *Y*-axis presents the contact angle values (CA) as the same function.

To clarify the surface activity of the synthesized SAILs and QASs, the values of CA and surface tension at CMC were correlated and plotted in Fig. 4. Interestingly, it can be observed that for AC14 and AC16 and their esterquats analogs, the CA and *γ*_CMC_ values were the highest. This trend is likely a response to a phenomenon that accompanies surfactants with long hydrophobic chains. The abovementioned amphiphilic part of the compounds could promote steric hindrance between surfactant molecules and, consequently, affect the formation of aggregates in aqueous solution.^45,51,52^

### 3.3. Foaming properties

When interpreting foaming properties, it is important to consider that foam formation is accompanied by the ordering of the compound molecules at the air–water interface. As is well known, surface-active compounds must adopt a lipid-like shape with the headgroup on the outside of the aggregate and the tail groups should be oriented toward the air at the air–water interface. At the same time, in addition to the aforementioned interpretation of potential molecular conformations of compounds, it is also necessary to focus on the chemical structure of the amidequats and the esterquats and their concentration.

**Fig. 5:**
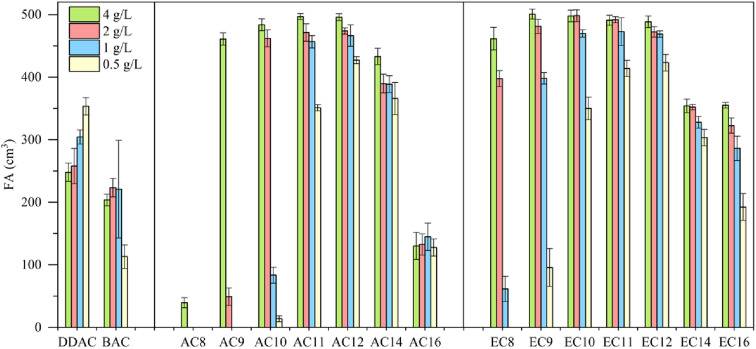
Foaming ability (FA) for amidequats and esterquats.

The foaming properties of the amidequats and the esterquats were tested at concentrations of 4, 2, 1 and 0.5 g L^−1^ and compared with the foaming properties of popular cationic surfactants, such as didecyldimethylammonium chloride (DDAC) and benzalkonium chloride (BAC). The results are shown in Fig. 5.

Compared to the amidequats, the esterquats generally show better foaming values, except for AC14, which showed a better foaming ability than that of its analog (EC14). In both groups of compounds, the influence of the alkyl chain length on the foaming properties is noticeable. The foaming ability increases with the length of the alkyl chain, reaches a maximum for the C12 substituent, and then decreases again. The concentration of the tested solutions exhibits a noticeable effect on the foaming properties, and the greatest differences are visible for compounds with shorter chains (C8–C10). In general, for all compounds, the foaming ability decreased with decreasing concentration. The exception is DDAC, for which the foaming ability increased with decreasing concentration. In the case of BAC for the first three concentrations (4, 2 and 1 g L^−1^), this ability remained at a comparable level, after which it decreased at the lowest concentration (0.5 g L^−1^). Compared to the esterquats, the amidequats with shorter alkyl chains (C8–C10) are more sensitive to concentration changes. Both esterquat and amidequat with a C12 substituent were more effective foaming compounds in the entire concentration range than classic cationic surfactants, such as DDAC and BAC. The foaming abilities of AC12 and EC12 were comparable. These compounds could be a new potential replacement for cocamidopropyl betaine, which is commonly used in hair shampoos and body wash gels as a washing and foaming compound.

**Fig. 6:**
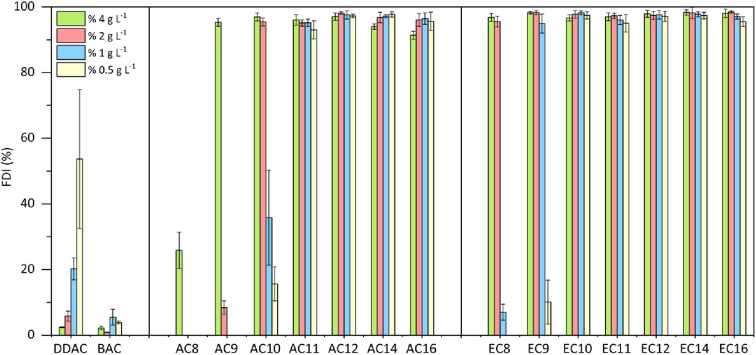
Foam durability index (FDI) for amidequats and esterquats.

The durability of the resulting foam was also tested, and the results are presented in Fig. 6. Differences in the durability of the resulting foam are visible for compounds with a shorter alkyl substituent (C8–C10); here, compared to the amidequats, the esterquats clearly show a more stable foam. For compounds with a substituent above C11, no significant differences in foam durability were observed, and it can be concluded that in the entire tested concentration range, these compounds show a high foam durability index > 91%. For compounds with a shorter substituent (C8–C10), foam durability decreased along with the dilution of the tested solutions. Only for the commercial compound DDAC did foam durability increase with lower concentration. In general, both reference substances (DDAC and BAC) showed very low values of foam durability in the tested concentration range, and both amidequats from the C11 to C16 substituent and esterquats from the C10 to C16 substituent are a clear competition for them.

### 3.4. Atomic force microscopy analysis

**Fig. 7:**
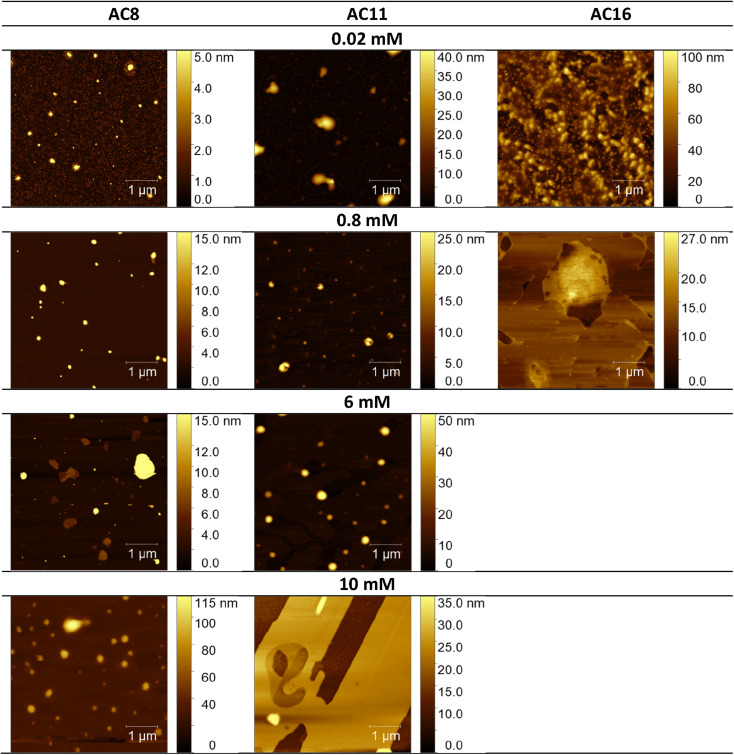
AFM studies of amidequats deposited on a mica surface. The surface coverage by amidequats molecules with different alkyl chain lengths and concentrations (topography of selected areas).

**Fig. 8:**
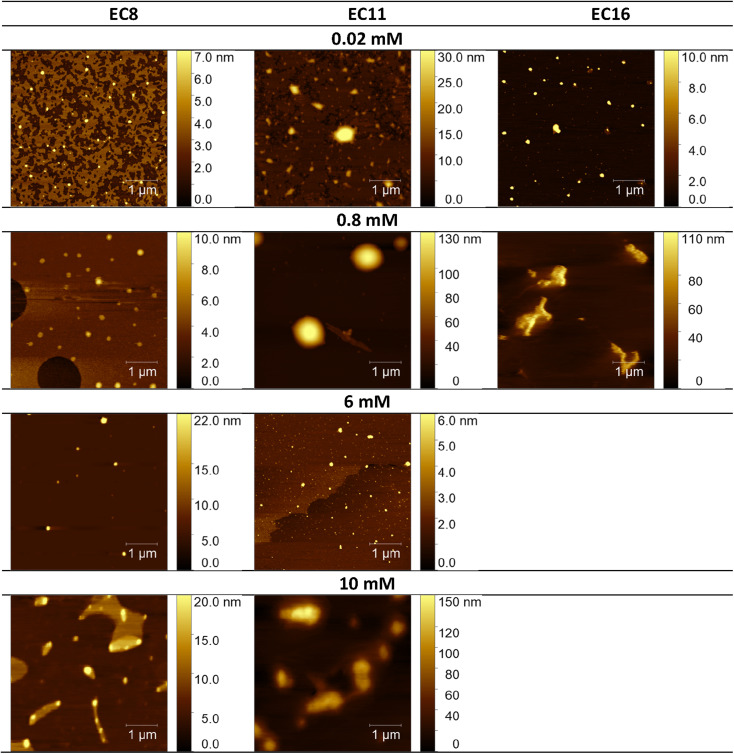
AFM studies of the esterquats deposited on a mica surface. The surface coverage by esterquats molecules with different alkyl chain lengths and concentrations (topography of selected areas).

**Fig. 9:**
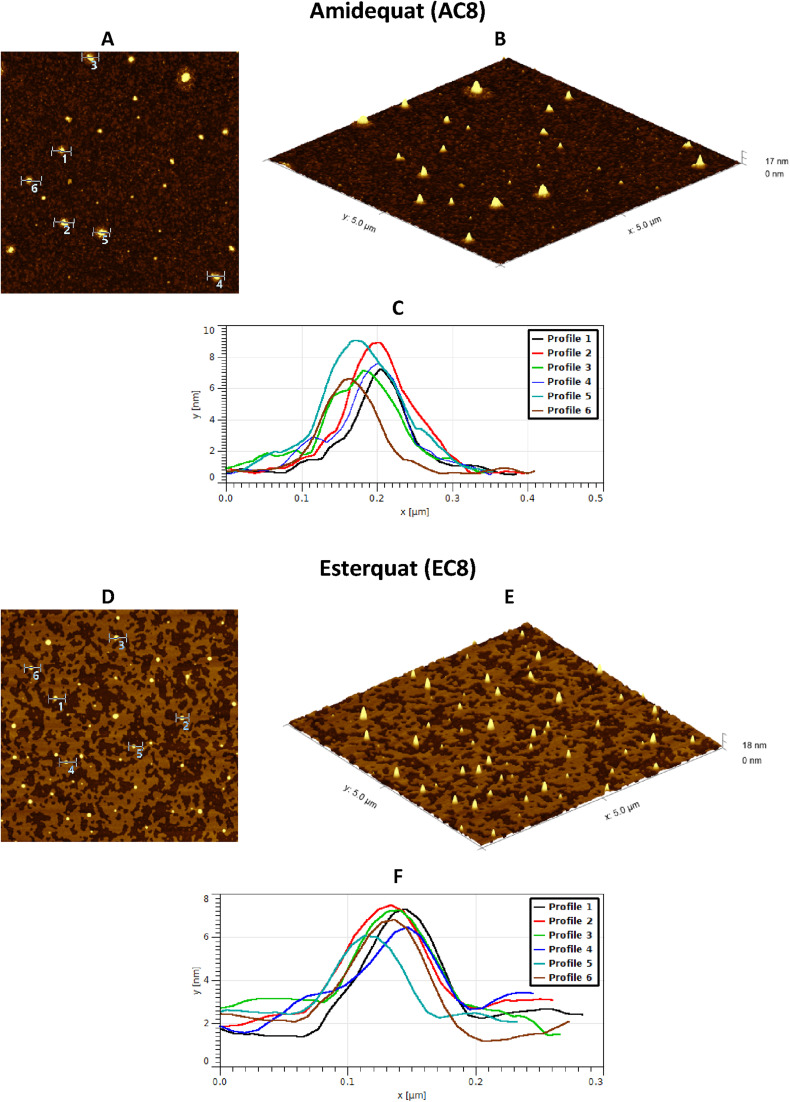
AFM results for the amidequat (AC8) and the esterquat (EC8), showing the difference with surface coverage. (A and D) Topography of selected areas of compounds. (B and E) 3D view of the test surfaces. (C and F) Six profile curves for selected round deposits. The concentration of the analyzed solution was 0.02 mM.

Considering the potential applications of the analyzed compounds as replacements for disinfectants or conventional surfactants, special attention should be focused on physical properties, including the ability of liquids to maintain contact with solid surfaces. Therefore, we used the atomic force microscopy technique to perform a topographical analysis of the sample surfaces (SAILs and QASs). This technique is used by many researchers who focus on studying the phenomenon of adsorption on different surfaces.^53–56^ The concentration of the solutions was chosen to focus on before CMC and after CMC concentrations of compounds. Fig. 7–9 present exemplary images of AFM studies.

Looking at the results, it is important to focus on several aspects, such as the elongation effects of the alkyl chain, the differences in structure between amidequats and esterquats, and the concentrations of the analyzed solutions. Generally, the samples seem to form spherical objects. Amidequats and esterquats are more likely to form vesicles, in the same way that phospholipids and two-tailed surfactants do. Moreover, this would correlate with some of the *ca.* 100 nm objects seen by AFM data and would also explain the sheet-like structures observed in surfactants with longer tails. Probably, these could be flat lamellar structures. Notably, at very low concentrations, the surface-active compounds adsorb as isolated molecules before forming small clusters.^31,34,57^ In addition, amidequat or esterquat molecules deposited on the mica surface form a flat spilled surface. The mentioned aggregates are observed when analyzing solutions above the CMC. The best mica surface coverage could be seen for AC16 and EC16. Consequently, the longer the alkyl chain is, the better the surface coverage.

According to our interpretation, the topography of the study area (see Fig. 9A and D) covered with the amidequat (AC8) shows a dense distribution of symmetric round deposits on the surface and is comparable to that observed for the esterquat (EC8). The mean symmetric round deposit sizes were 46.78 nm (AC8) and 45.61 nm (EC8), which confirm the abovementioned conclusion.

### 3.5. Antimicrobial activity studies

The antimicrobial activity of the analyzed compounds is summarized in Table 3. The aim of the study was to evaluate the effects of newly synthesized compounds on a broad range of microorganisms, with different cell structures, including Gram-positive and Gram-negative bacteria, fungi, and *Pseudomonas putida*, a soil bacterium known for its unique properties. Moreover, most of these strains are human opportunistic pathogens, causing a wide spectrum of life-threatening diseases and their use in the study was related to the constant need of novel substances exhibiting substantial activity against them. One of the focuses was to determine whether the new compounds could inhibit pathogenic bacteria without adversely affecting soil bacteria. *Pseudomonas putida* was chosen as the standard microorganism to evaluate the general ecotoxicity of the compounds. This could have significant implications for the development of new chemical compounds that can selectively target pathogenic bacteria without harming beneficial soil microorganisms.

**Table 3:** MIC values for the studied compounds

MIC (mmol L^−1^)								
Abbreviation		Human pathogenic microorganisms						Soil microorganism
		G(+)		G(−)			Fungi	G(−)
		SAU	EFA	PAE	ECO	KPN	CAL	PPT
**AMIDEQUATS**								
SAILs	AC8	0.098	0.098	0.761	0.393	3.068	0.196	0.761
	AC9	0.047	0.024	0.380	0.190	1.471	0.047	0.190
	AC10	0.011	0.011	0.367	0.092	0.712	0.011	0.184
	AC11	0.006	0.006	0.690	0.089	0.356	0.006	0.089
	AC12	0.011	0.005	0.669	0.086	0.345	0.005	0.173
	AC14	0.001	0.001	>10.170	0.631	>10.170	0.001	2.543
	AC16	0.019	0.010	>9.621	>9.621	>9.621	0.308	>9.621

**ESTERQUATS**								
QASs	EC8	0.024	0.049	0.759	0.196	0.759	0.024	0.759
	EC9	0.002	0.002	0.047	0.006	0.047	0.002	0.047
	EC10	0.023	0.011	0.367	0.092	0.367	0.006	0.183
	EC11	0.003	0.003	0.355	0.355	0.355	0.003	0.178
	EC12	0.011	0.011	2.691	0.344	1.335	0.011	0.667
	EC14	0.020	0.041	>10.150	2.537	>10.150	0.041	10.150
	EC16	0.010	0.010	>19.206	2.401	>19.206	0.038	>19.206
	BAC	0.028	0.014	1.765	0.057	0.456	0.014	0.459
	DDAC	0.004	0.007	0.856	0.014	0.856	0.007	0.442

Additionally, the MIC values of reference substances commonly used in disinfection and antiseptics, such as didecyldimethylammonium chloride (DDAC) and benzalkonium chloride (BAC), are presented with MIC values ranging from 0.004 to 1.765 mmol L^−1^.

The esterquats and the amidequats are types of structures with surface properties that exhibit antimicrobial activity against a range of microorganisms. This activity is based on their ability to disrupt the cell membrane of microorganisms, causing intracellular components to leak and ultimately leading to cell death. Generally, as seen in Table 3, the antimicrobial activity varies depending on the specific compound and microorganism tested. In general, both amidequats and esterquats demonstrate antimicrobial activity against a range of microorganisms. The amidequats show good activity against Gram-positive bacteria, such as *Staphylococcus aureus* (SAU) and *Enterococcus faecalis* (EFA), with MIC values ranging from 0.001 to 0.098 mmol L^−1^. However, their activity against Gram-negative bacteria, such as *Pseudomonas aeruginosa* (PAE), *Escherichia coli* (ECO), and *Klebsiella pneumoniae* (KPN), is generally less potent, with MIC values ranging from 0.086 to >10.170 mmol L^−1^. Similarly, the esterquats show better activity against Gram-positive bacteria, with MIC values ranging from 0.002 to 0.049 mmol L^−1^. When considering Gram-negative bacteria, the activity is considerably lower, with MIC values ranging from 0.006 to >19.206 mmol L^−1^. Moreover, both classes of compounds are active against fungi.

The table shows that esterquats with shorter chains (C8–C11) usually have lower MIC values for the tested human pathogens and fungi compared to esterquats with longer chains (C12 and higher). Moreover, it can be concluded that for shorter chains, esters are more active than amides. Notably, for compounds with longer chains (over C12), amidequats appear more active than esterquats. The cutoff point, *i.e.*, the point at which activity starts to decrease with longer chain lengths, is approximately C11 for esterquats and C12 for amidequats. However, for soil bacteria, the differences between esterquats and amidequats were not as significant.

Notably, the chain length in the molecule is only one factor that affect the activity of antibacterial and antifungal compounds. The occurrence of increased effectiveness at intermediate tail lengths in various cationic surfactant systems has been consistently observed previously, indicating a common pattern where specific tail lengths within these surfactants tend to demonstrate heightened activity or optimized performance.^58,59^

Overall, these results suggest that esterquats and amidequats have potential as antimicrobial agents, particularly against Gram-positive bacteria and fungi. The varying susceptibility of Gram-positive and Gram-negative bacteria ties back to their structural differences, especially in their cell membranes. Gram-positive bacteria have a simpler cell wall structure with a thicker peptidoglycan layer, which can make them more susceptible to certain substances that target this layer. On the other hand, Gram-negative bacteria have a more complex cell wall with an outer membrane that can act as a barrier, making it harder to penetrate and affect these bacteria.^58^ However, further studies are needed to determine the efficacy of these compounds and their potential in some industrial applications.

**Fig. 10:**
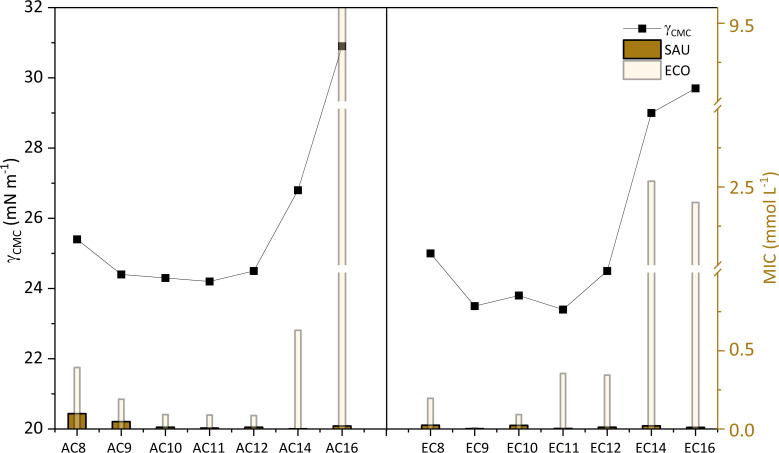
Influence of *γ*_CMC_ on MIC against Gram-positive bacteria (SAU), Gram-negative bacteria (ECO).

As reported in the literature,^58,60–63^ there is a close relationship between antimicrobial activity and properties characteristic for amphiphilic compounds. Especially when it comes to quaternary ammonium compounds, QACs^58,60,63,64^ which include amidequats and esterquats. An earlier section of this chapter described the role that alkyl substituents play in antimicrobial activity. Now, we will focus exclusively on presenting the relationship between antimicrobial activity and a selected surface property parameter such as *γ*_CMC_. For this purpose, Gram-positive bacteria (SAU), Gram-negative bacteria (ECO), and the MIC values obtained for them served in the discussion (see Fig. 10).

In Fig. 10 is presented, the correlation between the *γ*_CMC_ parameter with the antimicrobial activity. Interestingly, looking at the graph, it can be observed that for amidequats and esterquats the values of the individual parameters arrange themselves in the shape of a parabola. Compounds for which the *γ*_CMC_ values were the highest are observed to have the lowest antimicrobial activity. This is probably related to the increased hydrophobicity index to estimate the effectiveness of the analyzed compounds, and which is directly related to the permeability of the membranes of microorganisms. In the other hand, the second hypothesis involves a weaker interaction between amidequats and esterquats and microorganisms, which may be due to bacterial resistance.

## 4. Conclusion

An interesting strategy is synthesizing compounds based on natural resources, which was performed in this research. Amidequats and esterquats based on caprylic acid were synthesized in high yield (≥81%) and high purities (≥92%), and their structures were confirmed by ^1^H NMR and ^13^C NMR. By analyzing the melting point, we classified the tested salts into the following groups: SAILs and QASs.

Due to their amphiphilic structure, amidequats and esterquats are surface-active compounds. Moreover, the surface property results indicated that the studied SAILs and QASs successfully decrease the surface tension ranged from 23.4 to 30.9 mN m^−1^, which is lower than that achieved with the cationic surfactant BAC and approximately to that of DDAC.^37^ Through a comprehensive study of surface activity, we concluded that the introduction of ester or amide groups in the substituent located in the amphiphilic cation leads to higher surface activity than that of conventional surfactants, namely, homologous series of alkyltrimethylammonium bromides^39–43^ and domiphen bromide,^38^ and the new amidequats and esterquats were classified as unique compared to other surfactants.

Topography analyses showed that as the alkyl chain lengthens, the degree of surface coverage increases. In general, compared to the amidequats, the esterquats show better foaming values. Both esterquat and amidequat with a C12 substituent were more effective foaming compounds in the entire concentration range than classic cationic surfactants, such as DDAC and BAC. The foaming ability and foam durability index of AC12 and EC12 were comparable.

The results obtained by microbiological tests demonstrated that for shorter chains, esterquats are more active than amidequats. Notably, for compounds with longer chains (over C12), the amidequats exhibit greater antimicrobial activity than that of the esterquats. The cutoff point is approximately C11 for the esterquats and C12 for the amidequats. However, for soil bacteria, the differences between the esterquats and the amidequats were not as significant.

In summary, it has been shown that amidequat and esterquat with twelve carbon atoms in the alkyl chain can be potentially used as very good foaming agents. On the other hand, when looking for the most prosperous disinfectant that also acts as a surface-active agent, no particular compound can be clearly singled out. However, the compounds with a short alkyl chain (to eleven carbon atoms in the alkyl substituent) may be the most prosperous and can be used in this case as multifunctional compounds.

The key issue is that the entire series of compounds studied show a range of potential applications, which corresponds with the concept of sustainable development. Consequently, these discoveries may help provide opportunities to expand the field of green chemistry to include a family of potentially alternative groups that are stronger than conventional cationic surfactants.

## Conflicts of interest

The authors declare no conflicts of interest.

## Supplementary Material

RA-013-D3RA07127H-s001
