# Supplementary material for: Quaternary ammonium salts based on caprylic acid as antimicrobial and surface-active agents
Source: RSC Adv. 2023 Nov 29;13(49):34782–97. doi: 10.1039/d3ra07127h (PMC10685092; doi:10.1039/d3ra07127h)
Supplement: RA-013-D3RA07127H-s001 [file RA-013-D3RA07127H-s001.pdf]

Electronic Supplementary Material (ESI) for RSC Advances.
This journal is © The Royal Society of Chemistry 2023

Quaternary ammonium salts based on caprylic acid as antimicrobial and surface-active agents

Marta Wojcieszak [a], Anna Syguda [a], Maciej Karolak [b], Łukasz Pałkowski [b],
Katarzyna Materna [a,]*

[a] Poznan University of Technology, Faculty of Chemical Technology, Berdychowo 4, 60-965, Poznan, Poland.

[b] Department of Pharmaceutical Technology, Faculty of Pharmacy, Nicolaus Copernicus University, Jurasza 2, 85-089 Bydgoszcz, Poland.

* corresponding author: e-mail: katarzyna.materna@put.poznan.pl

List of tables

Table S1. Chemical shifts (δ) and coupling constants (*J*) values in ^{1}H NMR spectra for aminoamide and aminoester based on caprylic acid ($CDCl_3$). 5
Table S2. Chemical shift (δ) values in ^{13}C NMR spectra for aminoamide and aminoester based on caprylic acid ($CDCl_3$). 6
Table S3. Chemical shifts (δ) and coupling constants (*J*) values in ^{1}H NMR spectra for amidequats based on caprylic acid ($CDCl_3$). 7
Table S4. Chemical shift (δ) values in ^{13}C NMR spectra for amidequats based on caprylic acid ($CDCl_3$). 8
Table S5. Chemical shifts (δ) and coupling constants (*J*) values in ^{1}H NMR spectra for esterquats based on caprylic acid ($CDCl_3$). 9
Table S6. Chemical shift (δ) values in ^{13}C NMR spectra for esterquats based on caprylic acid ($CDCl_3$). 10

List of figures

Fig. S1. ^{1}H NMR spectrum of *N*-[(2-dimethylamino)ethyl]octanamide. 11
Fig. S2. ^{13}C NMR spectrum of *N*-[(2-dimethylamino)ethyl]octanamide. 12
Fig. S3. ^{1}H NMR spectrum of (2-dimethylamino)ethyl octanate. 13
Fig. S4. ^{13}C NMR spectrum of (2-dimethylamino)ethyl octanate. 14
Fig. S5. ^{1}H NMR spectrum of dimethyl-*N*-[(2-octanamido)ethyl]octylammonium bromide (**AC8**). 15
Fig. S6. ^{13}C NMR spectrum of dimethyl-*N*-[(2-octanamido)ethyl]octylammonium bromide (**AC8**). 16
Fig. S7. ^{1}H NMR spectrum of dimethylnonyl-*N*-[(2-octanamido)ethyl]ammonium bromide (**AC9**). 17
Fig. S8. ^{13}C NMR spectrum of dimethylnonyl-*N*-[(2-octanamido)ethyl]ammonium bromide (**AC9**). 18
Fig. S9. ^{1}H NMR spectrum of decyldimethyl-*N*-[(2-octanamido)ethyl]ammonium bromide (**AC10**). 19

Fig. S10. ^{13}C NMR spectrum of decyldimethyl-*N*-[(2-octanamido)ethyl]ammonium bromide (**AC10**). 20
Fig. S11. ^{1}H NMR spectrum of dimethyl-*N*-[(2-octanamido)ethyl]undecylammonium bromide (**AC11**). 21
Fig. S12. ^{13}C NMR spectrum of dimethyl-*N*-[(2-octanamido)ethyl]undecylammonium bromide (**AC11**). 22
Fig. S13. ^{1}H NMR spectrum of dodecyldimethyl-*N*-[(2-octanamido)ethyl]ammonium bromide (**AC12**). 23
Fig. S14. ^{13}C NMR spectrum of dodecyldimethyl-*N*-[(2-octanamido)ethyl]ammonium bromide (**AC12**) 24
Fig. S15. ^{1}H NMR spectrum of dimethyl-*N*-[(2-octanamido)ethyl]tetradecylammonium bromide (**AC14**) 25
Fig. S16. ^{13}C NMR spectrum of dimethyl-*N*-[(2-octanamido)ethyl]tetradecylammonium bromide (**AC14**) 26
Fig. S17. ^{1}H NMR spectrum of hexadecyldimethyl-*N*-[(2-octanamido)ethyl]ammonium bromide (**AC16**) 27
Fig. S18. ^{13}C NMR spectrum of hexadecyldimethyl-*N*-[(2-octanamido)ethyl]ammonium bromide (**AC16**) 28
Fig. S19. ^{1}H NMR spectrum of dimethyl-2-octanoyloxyethyloctylammonium bromide (**EC8**). 29
Fig. S20. ^{13}C NMR spectrum of dimethyl-2-octanoyloxyethyloctylammonium bromide (**EC8**). 30
Fig. S21. ^{1}H NMR spectrum of dimethylnonyl-2-octanoyloxyethylammonium bromide (**EC9**). 31
Fig. S22. ^{13}C NMR spectrum of dimethylnonyl-2-octanoyloxyethylammonium bromide (**EC9**). 32
Fig. S23. ^{1}H NMR spectrum of decyldimethyl-2-octanoyloxyethylammonium bromide (**EC10**). 33
Fig. S24. ^{13}C NMR spectrum of decyldimethyl-2-octanoyloxyethylammonium bromide (**EC10**). 34
Fig. S25. ^{1}H NMR spectrum of dimethyl-2-octanoyloxyethylundecylammonium bromide (**EC11**). 35

Fig. S26. ^{13}C NMR spectrum of dimethyl-2-octanoyloxyethylundecylammonium bromide (**EC11**)....................36
Fig. S27. ^{1}H NMR spectrum of dodecyldimethyl-2-octanoyloxyethylammonium bromide (**EC12**)....................37
Fig. S28. ^{13}C NMR spectrum of dodecyldimethyl-2-octanoyloxyethylammonium bromide (**EC12**)....................38
Fig. S29. ^{1}H NMR spectrum of dimethyl-2-octanoyloxyethyltetradecylammonium bromide (**EC14**)....................39
Fig. S30. ^{13}C NMR spectrum of dimethyl-2-octanoyloxyethyltetradecylammonium bromide (**EC14**)....................40
Fig. S31. ^{1}H NMR spectrum of hexadecyldimethyl-2-octanoyloxyethylammonium bromide (**EC16**)....................41
Fig. S32. ^{13}C NMR spectrum of hexadecyldimethyl-2-octanoyloxyethylammonium bromide (**EC16**)....................42
Fig. S33. Effect of the elongation of alkyl chain on the CMC of amidequats and esterquats….43

Table S1. Chemical shifts (δ) and coupling constants (*J*) values in ^{1}H NMR spectra for aminoamide and aminoester based on caprylic acid ($CDCl_3$).

Protons	Number of protons	Multiplicity of the signal	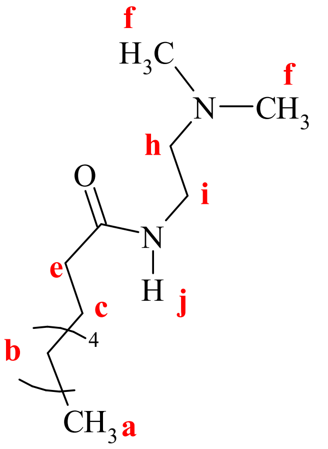		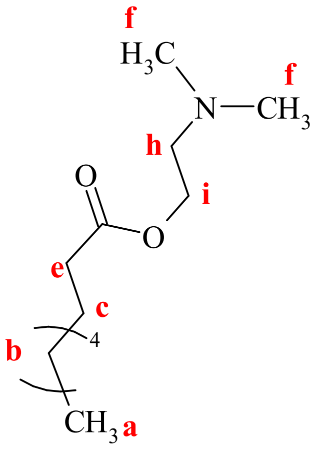	
			δ (ppm)	*J* (Hz)	δ (ppm)	*J* (Hz)
**a**	3H	triplet	0.82	6.6	0.87	6.8
**b**	2nH	multiplet	1.24	-	1.27	-
**c**	2H	quintet	1.57	7.0	1.61	7.0
**e**	2H	triplet	2.12	7.7	2.31	7.5
**f**	6H	singlet	2.19	-	2.27	-
**h**	2H	triplet	2.37	6.0	2.55	5.7
**i**	2H	quartet/ triplet	3.28 (quartet)	5.7	4.16 (triplet)	5.7
**j**	1H	triplet	6.13	5.1	-	-

Table S2. Chemical shift (δ) values in ^{13}C NMR spectra for aminoamide and aminoester based on caprylic acid ($CDCl_3$).

Carbon atoms	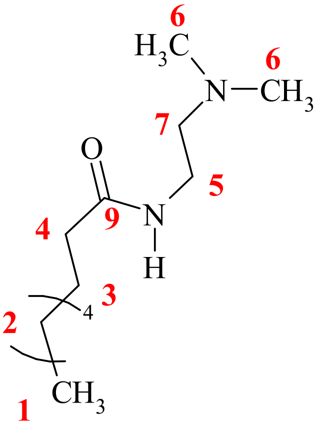	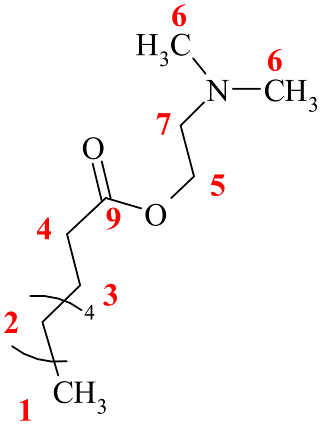
	δ (ppm)	δ (ppm)
**1**	13.95	13.99
**2**	22.49, 25.69, 28.92, 29.17	22.52, 24.87, 28.86, 29.02
**3**	31.60	31.59
**4**	36.55	34.19
**5**	36.62	61.94
**6**	44.99	45.64
**7**	57.81	57.79
**9**	173.21	173.86

Table S3. Chemical shifts (δ) and coupling constants (*J*) values in ^{1}H NMR spectra for amidequats based on caprylic acid ($CDCl_3$).

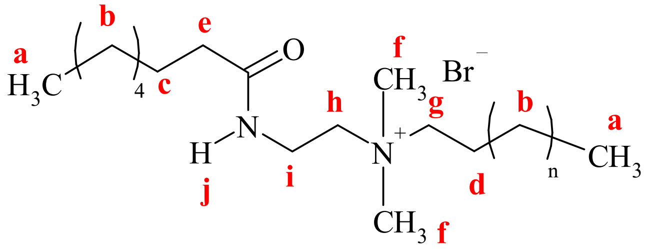

Protons	Number of protons	Multiplicity of the signal	Compound													
			AC8		AC9		AC10		AC11		AC12		AC14		AC16	
			n = 5		n = 6		n = 7		n = 8		n = 9		n = 11		n = 13	
			δ (ppm)	*J* (Hz)	δ (ppm)	*J* (Hz)	δ (ppm)	*J* (Hz)	δ (ppm)	*J* (Hz)	δ (ppm)	*J* (Hz)	δ (ppm)	*J* (Hz)	δ (ppm)	*J* (Hz)
**a**	6H	triplet	0.82	6.8	0.83	6.9	0.83	6.6	0.83	6.8	0.83	6.6	0.82	6.9	0.88	7.0
**b**	2(4+n)H	multiplet	1.21, 1.30	-	1.21, 1.30	-	1.21, 1.31	-	1.20, 1.30	-	1.21, 1.31	-	1.20, 1.30	-	1.25, 1.36	-
**c**	2H	quintet	1.55	7.2	1.56	7.1	1.56	6.9	1.56	7.2	1.56	6.9	1.56	6.9	1.62	7.4
**d**	2H	multiplet	1.69	-	1.69	-	1.70	-	1.69	-	1.69	-	1.68	-	1.70	-
**e**	2H	triplet	2.21	7.7	2.22	7.7	2.23	7.7	2.22	7.8	2.23	7.7	2.22	7.7	2.22	7.7
**f**	6H	singlet	3.32	-	3.32	-	3.32	-	3.33	-	3.33	-	3.32	-	3.32	-
**g**	2H	triplet	3.51	8.5	3.52	8.5	3.50	8.4	3.52	8.6	3.51	8.3	3.51	8.4	3.50	8.6
**h**	2H	multiplet	3.70	-	3.71	-	3.71	-	3.71	-	3.72	-	3.71	-	3.77	-
**i**	2H															
**j**	1H	triplet	8.23	5.0	8.23	5.0	8.25	5.1	8.23	5.0	8.25	5.1	8.23	5.1	8.28	5.0

Table S4. Chemical shift (δ) values in ^{13}C NMR spectra for amidequats based on caprylic acid ($CDCl_3$).

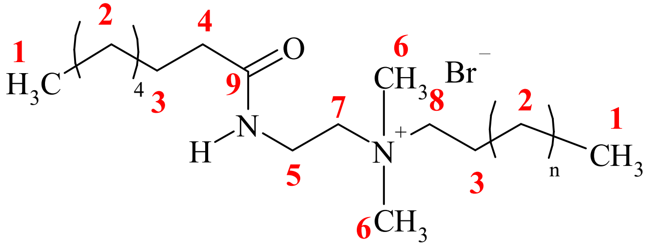

Carbon atoms	AC8	AC9	AC10	AC11	AC12	AC14	AC16
	n = 5	n = 6	n = 7	n = 8	n = 9	n = 11	n = 13
	δ (ppm)	δ (ppm)	δ (ppm)	δ (ppm)	δ (ppm)	δ (ppm)	δ (ppm)
**1**	13.86, 13.90	13.91	13.96	13.92, 13.94	13.96, 13.99	13.93, 13.96	14.09
**2**	22.38, 22.43, 22.69, 25.31, 26.09, 28.87, 28.89, 28.99, 29.16	22.45, 22.71, 25.33, 26.11, 28.90, 28.95, 29.05, 29.17, 29.18	22.50, 22.52, 22.75, 25.39, 26.16, 28.94, 29.10, 29.21, 29.28, 29.29	22.46, 22.50, 22.72, 25.34, 26.12, 28.92, 29.08, 29.12, 29.18, 29.25, 29.31, 29.37	22.50, 22.55, 22.75, 25.39, 26.17, 28.95, 29.11, 29.20, 29.21, 29.29, 29.35, 29.46	22.48, 22.53, 22.74, 25.35, 26.14, 28.92, 29.10, 29.20, 29.27, 29.33, 29.45, 29.49, 29.52	22.62, 22.67, 22.85, 25.48, 26.24, 29.17, 29.33, 29.43, 29.56, 29.65
**3**	31.45, 31.53	31.54, 31.59	31.59, 31.70	31.56, 31.70	31.60, 31.76	31.56, 31.76	31.81, 31.90
**4**	33.85	33.86	33.89	33.87	33.92	33.89	34.05
**5**	36.15	36.17	36.19	36.17	36.20	36.17	36.34
**6**	51.56	51.57	51.60	51.57	51.61	51.58	51.74
**7**	62.47	62.49	62.56	62.50	62.56	62.52	62.88
**8**	65.39	65.41	65.49	65.42	65.50	65.44	65.86
**9**	174.59	174.61	174.75	174.62	174.72	174.64	174.91

Table S5. Chemical shifts (δ) and coupling constants (*J*) values in ^{1}H NMR spectra for esterquats based on caprylic acid ($CDCl_3$).

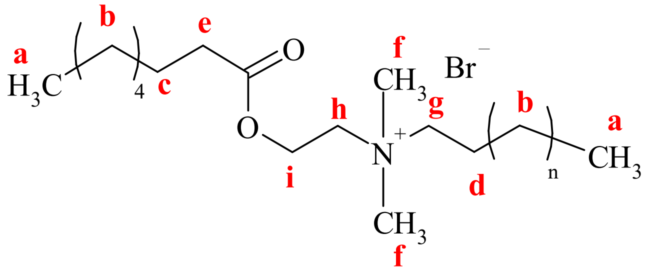

Protons	Number of protons	Multiplicity of the signal	Compound													
			EC8		EC9		EC10		EC11		EC12		EC14		EC16	
			m = 5		m = 6		m = 7		m = 8		m = 9		m = 11		m = 13	
			δ (ppm)	*J* (Hz)	δ (ppm)	*J* (Hz)	δ (ppm)	*J* (Hz)	δ (ppm)	*J* (Hz)	δ (ppm)	*J* (Hz)	δ (ppm)	*J* (Hz)	δ (ppm)	*J* (Hz)
**a**	6H	triplet	0.88	6.4	0.88	6.5	0.88	6.4	0.88	6.7	0.88	6.7	0.88	6.7	0.88	6.8
**b**	2(4+n)H	multiplet	1.28, 1.36	-	1.27, 1.36	-	1.26, 1.36	-	1.26, 1.36	-	1.26, 1.36	-	1.26, 1.36	-	1.26, 1.35	-
**c**	2H	quintet	1.61	7.2	1.61	7.2	1.61	7.2	1.61	7.3	1.61	7.2	1.61	7.2	1.61	7.0
**d**	2H	multiplet	1.78	-	1.78	-	1.77	-	1.77	-	1.77	-	1.77	-	1.76	-
**e**	2H	triplet	2.35	7.6	2.35	7.7	2.35	7.6	2.35	7.7	2.35	7.7	2.35	7.7	2.35	7.6
**f**	6H	singlet	3.49	-	3.50	-	3.48	-	3.51	-	3.50	-	3.51	-	3.49	-
**g**	2H	triplet	3.65	8.4	3.63	8.5	3.59	8.4	3.62	8.5	3.63	8.5	3.62	8.5	3.58	8.4
**h**	2H	triplet	4.08	4.6	4.09	4.8	4.05	4.6	4.09	4.8	4.09	4.7	4.09	4.7	4.07	4.8
**i**	2H	triplet	4.58	4.4	4.58	4.6	4.58	4.5	4.57	4.6	4.58	4.5	4.57	4.6	4.57	4.7

Table S6. Chemical shift (δ) values in ^{13}C NMR spectra for esterquats based on caprylic acid ($CDCl_3$).

Carbon atoms	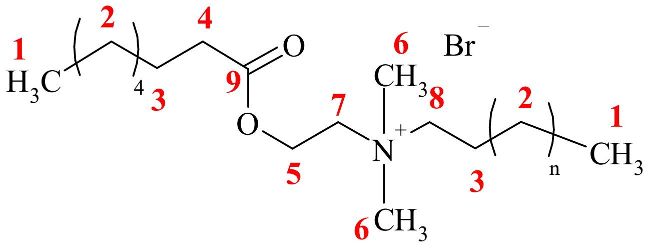						
	**Compound**						
	**EC8**	**EC9**	**EC10**	**EC11**	**EC12**	**EC14**	**EC16**
	n = 5	n = 6	n = 7	n = 8	n = 9	n = 11	n = 13
	δ (ppm)	δ (ppm)	δ (ppm)	δ (ppm)	δ (ppm)	δ (ppm)	δ (ppm)
**1**	13.74	13.86, 13.89	13.92, 13.96	19.91, 13.97	13.84, 13.90	13.91, 13.98	13.96, 14.02
**2**	22.26, 22.66, 24.37, 26.00, 28.58, 28.77, 28.78, 28.92,	22.38, 22.43, 22.78, 24.49, 26.12, 28.71, 28.89, 28.95, 29.21	22.45, 22.52, 22.82, 24.55, 26.19, 28.78, 28.95, 29.12, 29.16, 29.32	22.44, 22.53, 22.83, 24.55, 26.19, 28.77, 28.95, 29.16, 29.31, 29.36, 29.41	22.37, 22.46, 22.76, 24.47, 26.12, 28.69, 28.87, 29.11, 29.25, 29.29, 29.39	22.45, 22.55, 22.83, 24.55, 26.20, 28.77, 28.95, 29.17, 29.22, 29.32, 29.37, 29.47, 29.52, 29.55	22.48, 22.59, 22.80, 22.86, 24.72, 26.20, 28,81, 28.98, 29.15, 29.21, 29.26, 29.33, 29.37, 29.41, 29.52, 29.57, 29,60
**3**	31.31, 31.34	31.44, 31.58	31.49, 31.70	31.49, 31.74	31.41, 31.68	31.49, 31.78	31.53, 31.82
**4**	33.75	33.87	33.94	33.93	33.86	33.93	33.96
**5**	61.90	62.00	62.08	62.03	61.97	62.04	62.11
**6**	51.62	51.73	51.86	51.78	51.71	51.79	52.02
**7**	57.43	57.49	57.60	57.51	57.49	57.52	57.54
**8**	65.17	65.26	65.31	65.30	65.22	65.30	65.37
**9**	172.44	172.57	172.67	172.62	172.55	127.63	172.68

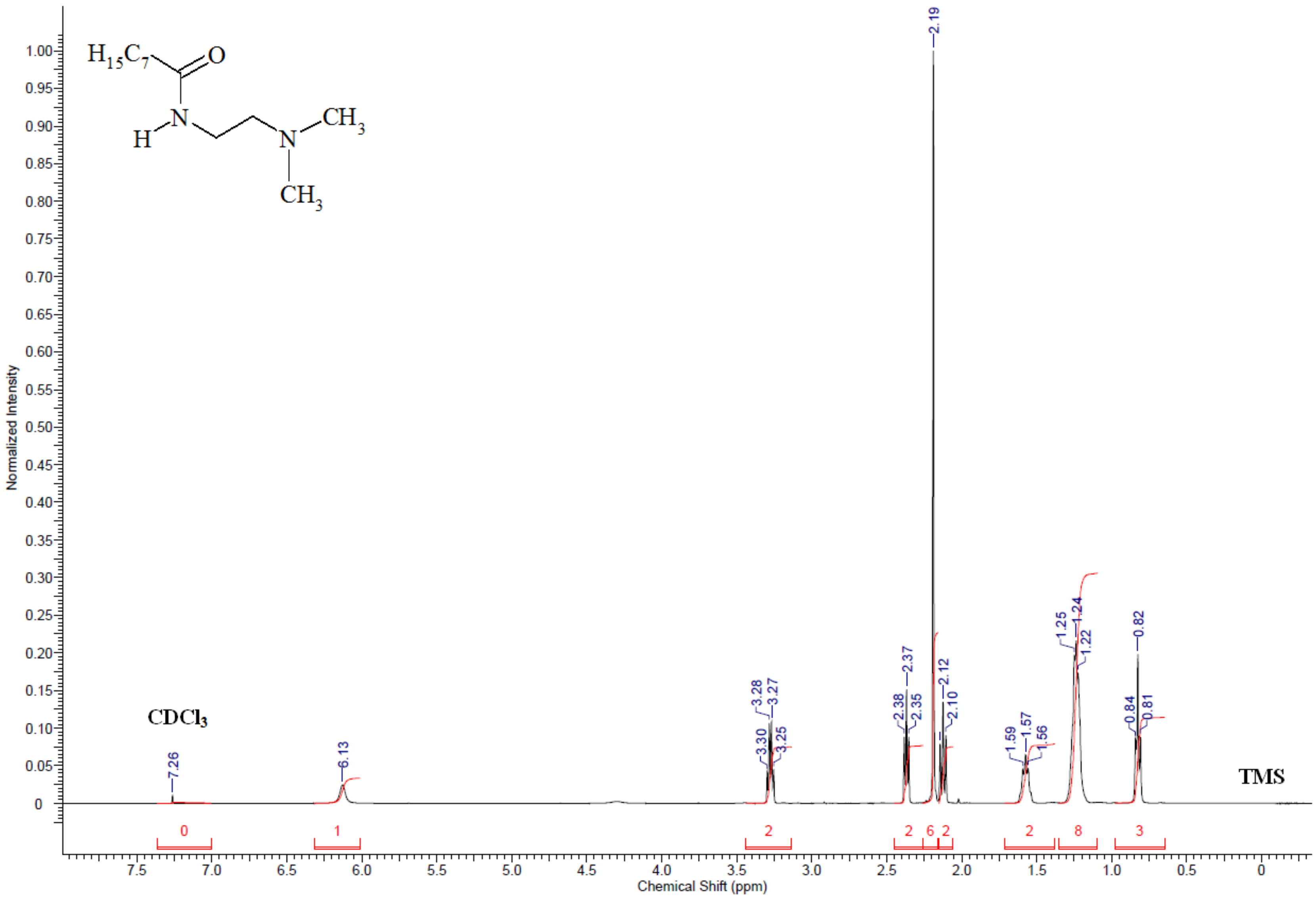

Fig. S1. ^{1}H NMR spectrum of *N*-[(2-dimethylamino)ethyl]octanamide.

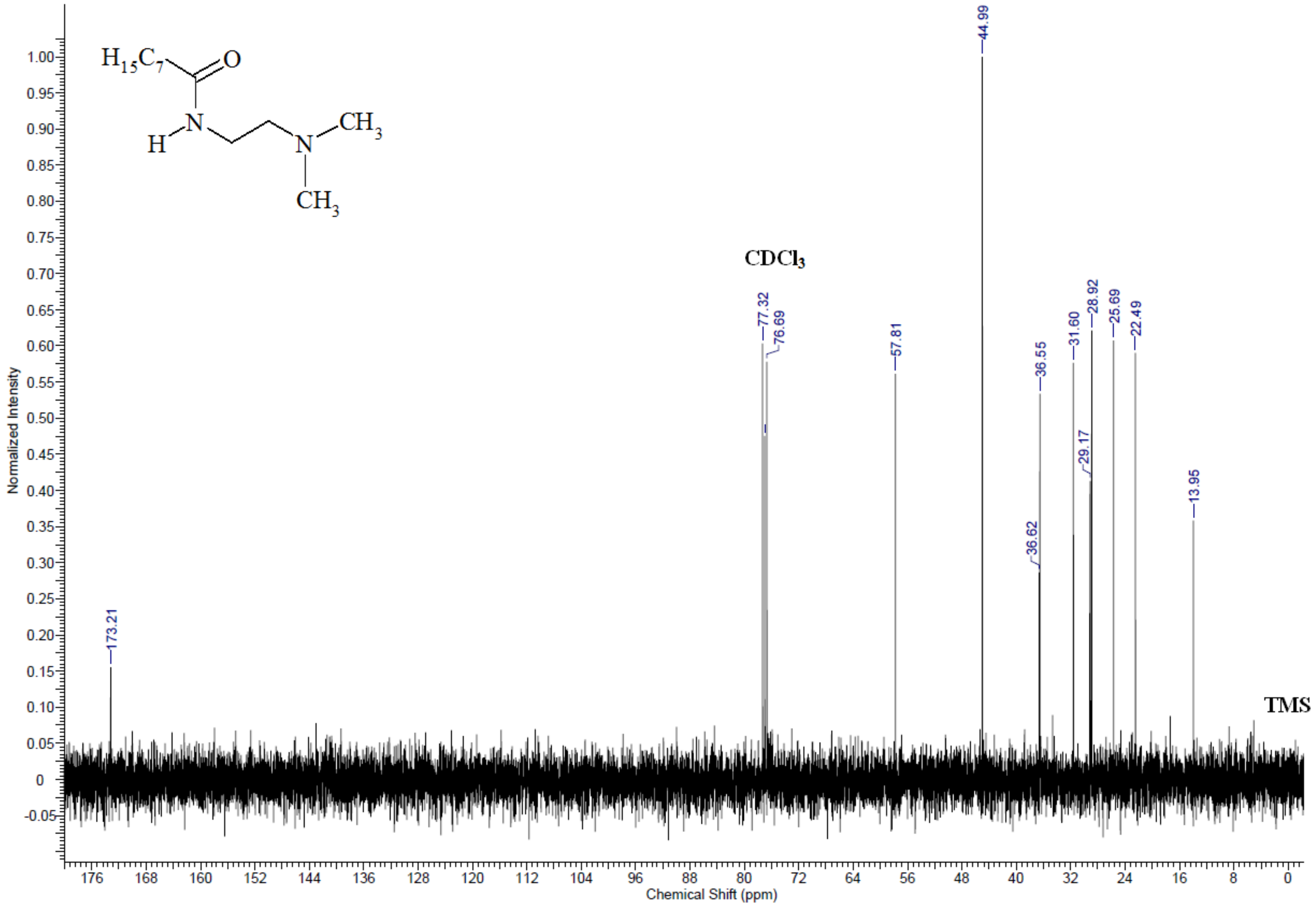

Fig. S2. ^{13}C NMR spectrum of *N*-[(2-dimethylamino)ethyl]octanamide.

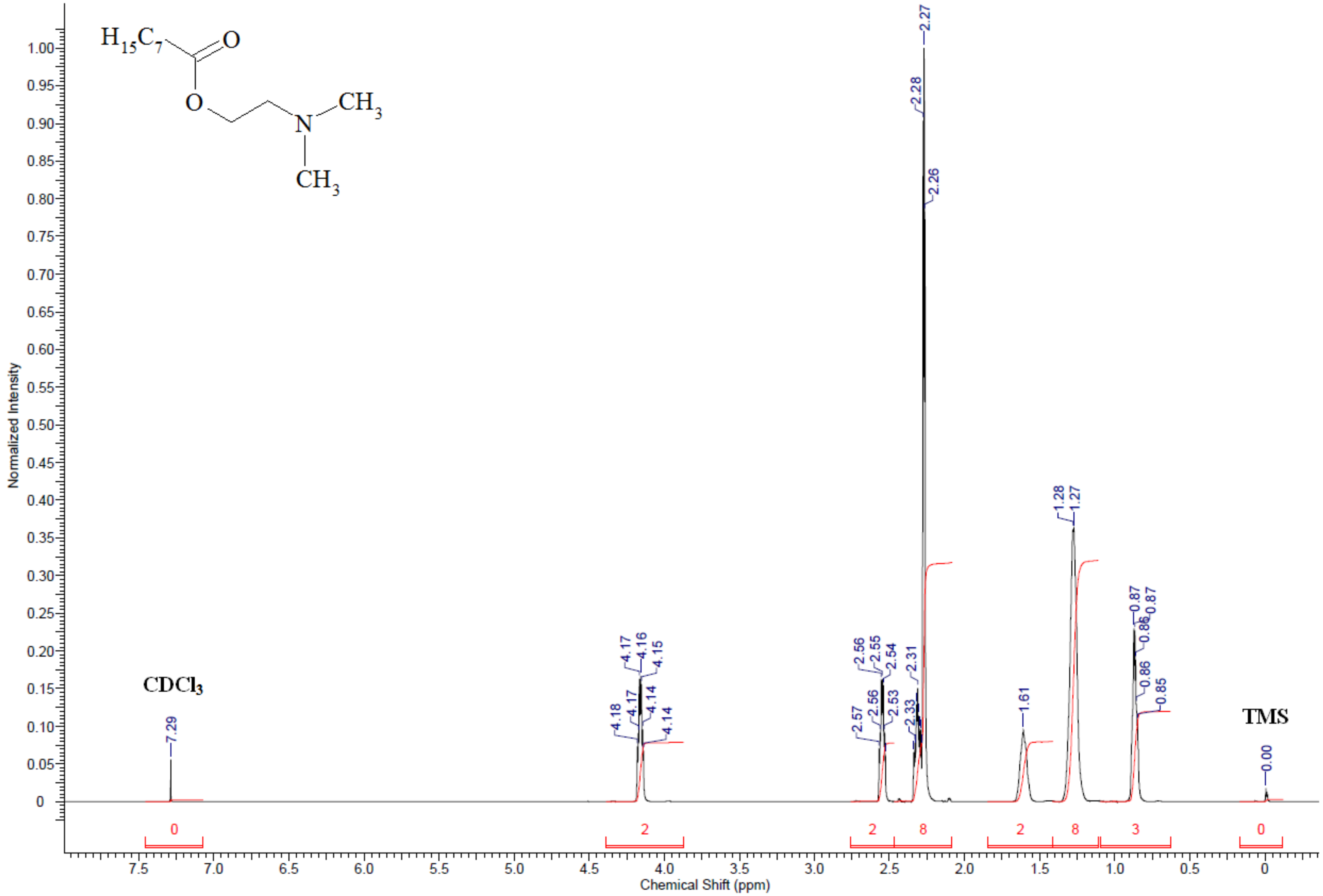

Fig. S3. ^{1}H NMR spectrum of (2-dimethylamino)ethyl octanate.

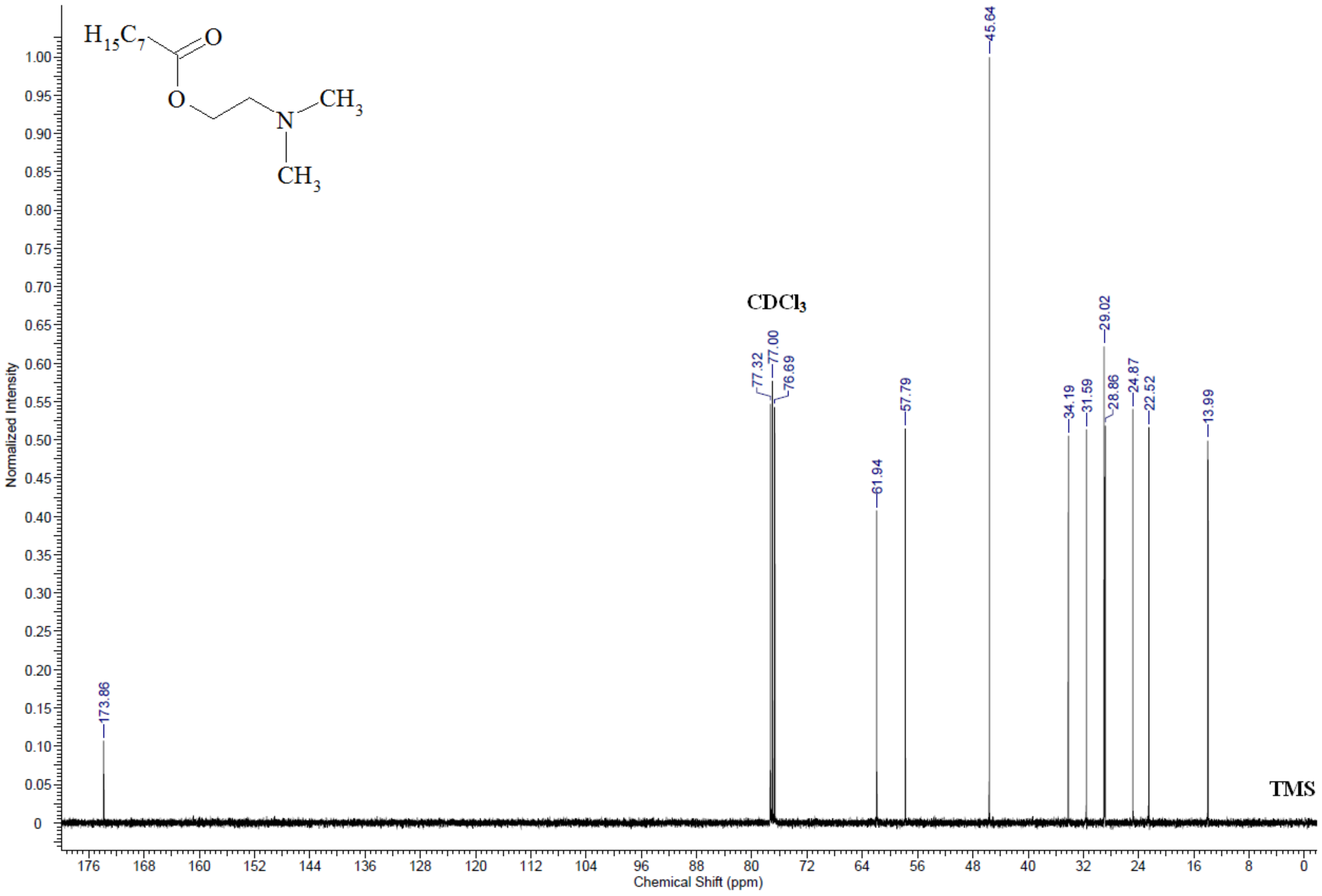

Fig. S4. ^{13}C NMR spectrum of (2-dimethylamino)ethyl octanate.

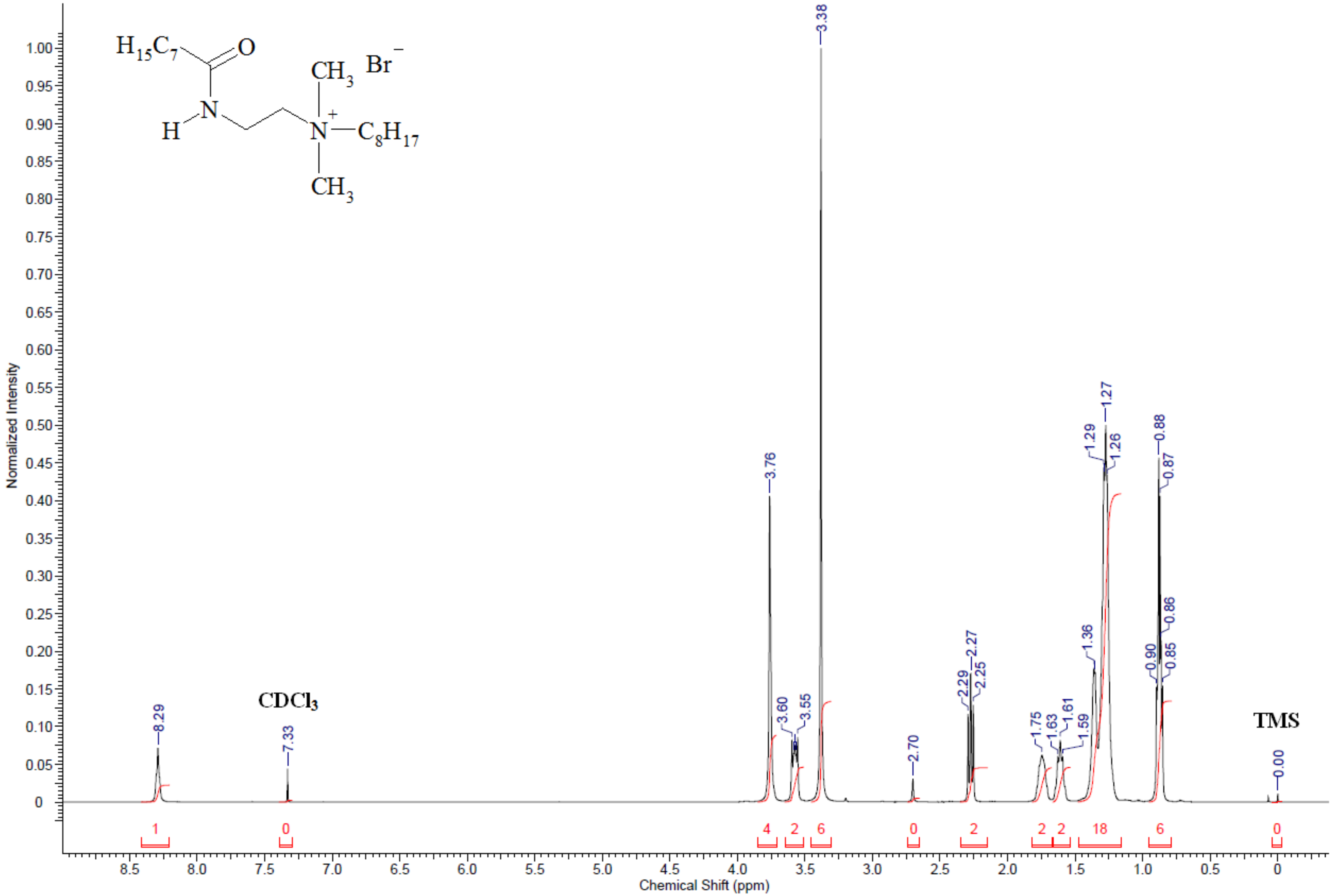

Fig. S5. ^{1}H NMR spectrum of dimethyl-*N*-[(2-octanamido)ethyl]octylammonium bromide (**AC8**).

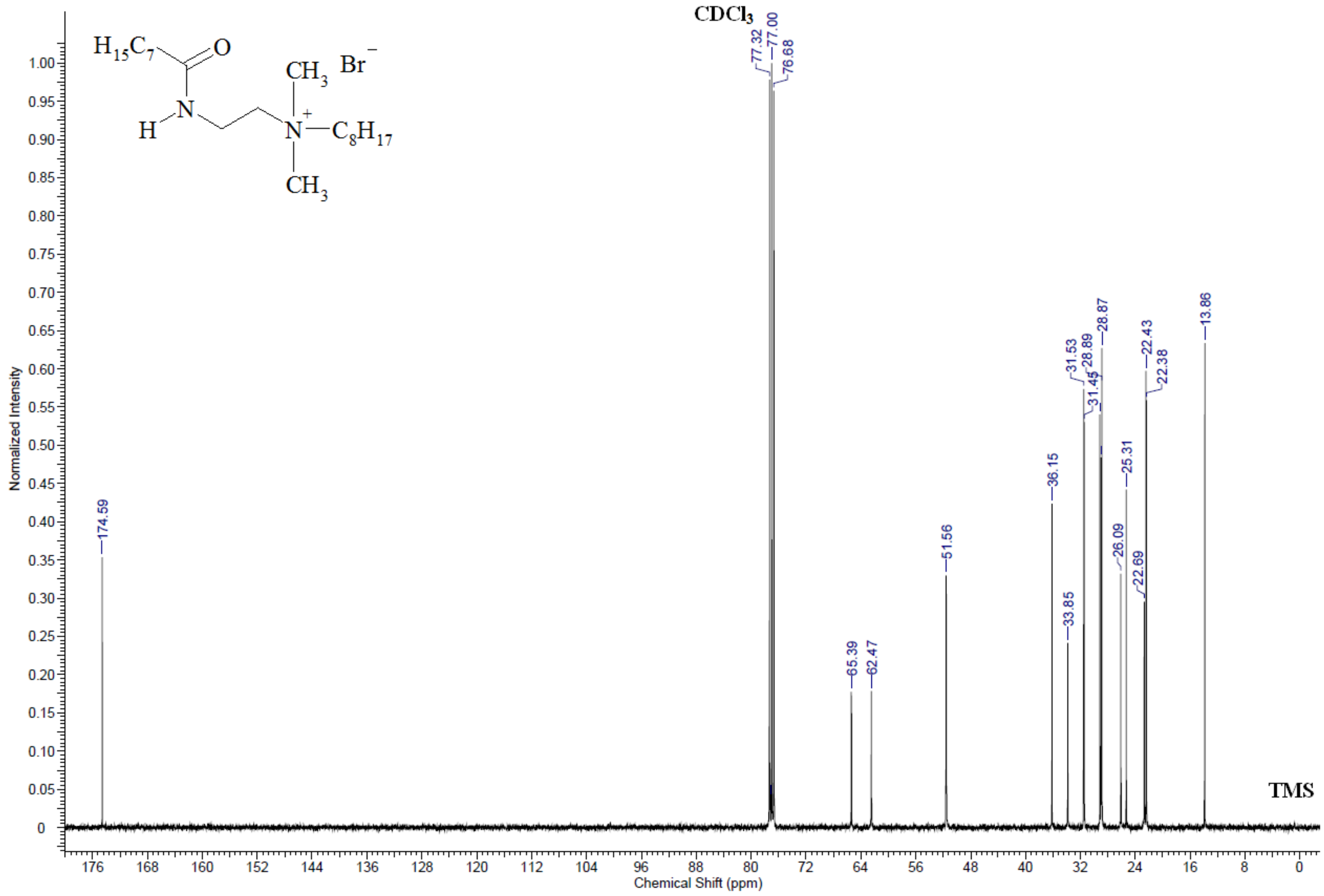

Fig. S6.^{13}C NMR spectrum of dimethyl-*N*-[(2-octanamido)ethyl]octylammonium bromide (**AC8**).

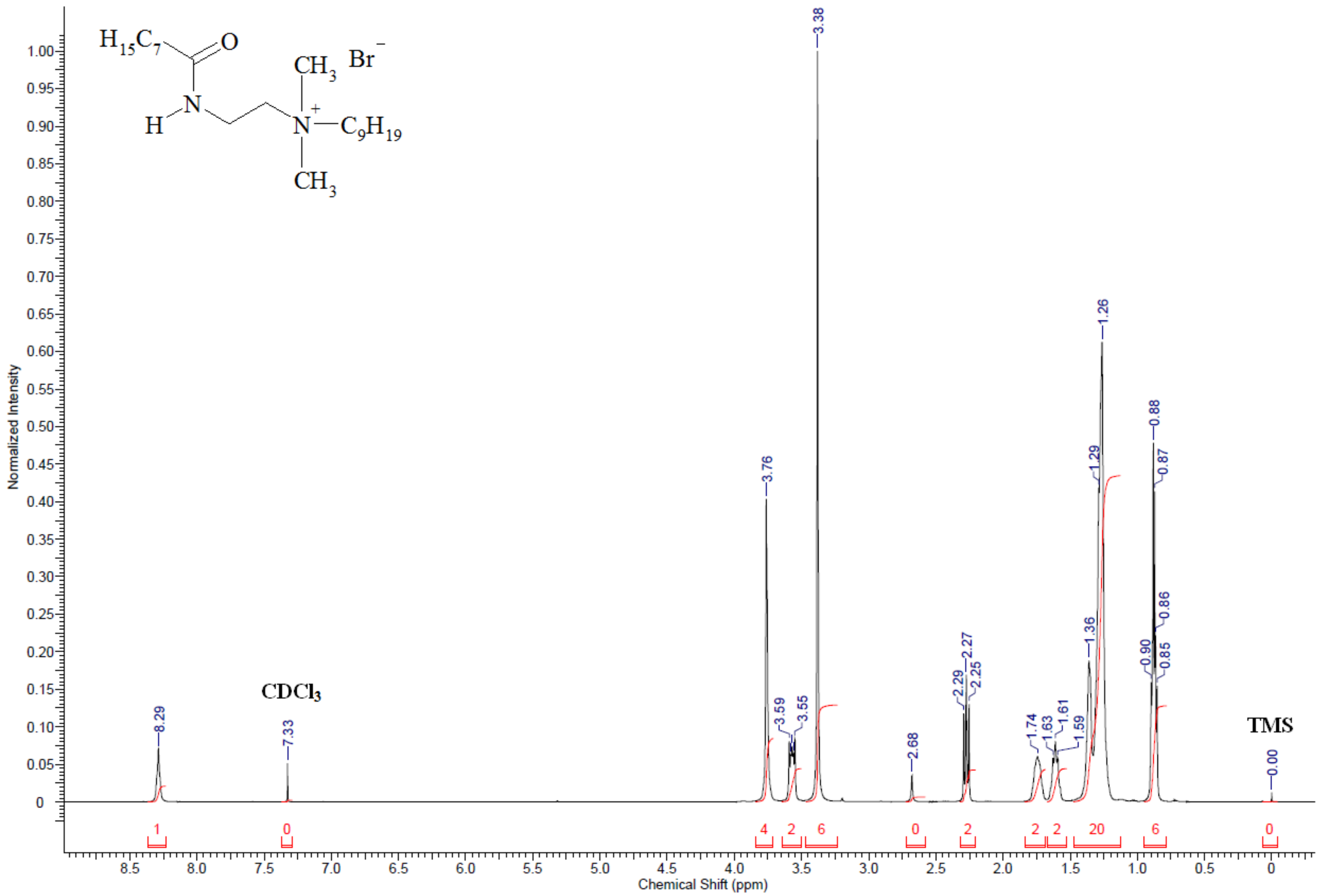

Fig. S7. ^{1}H NMR spectrum of dimethylnonyl-*N*-[(2-octanamido)ethyl]ammonium bromide (**AC9**).

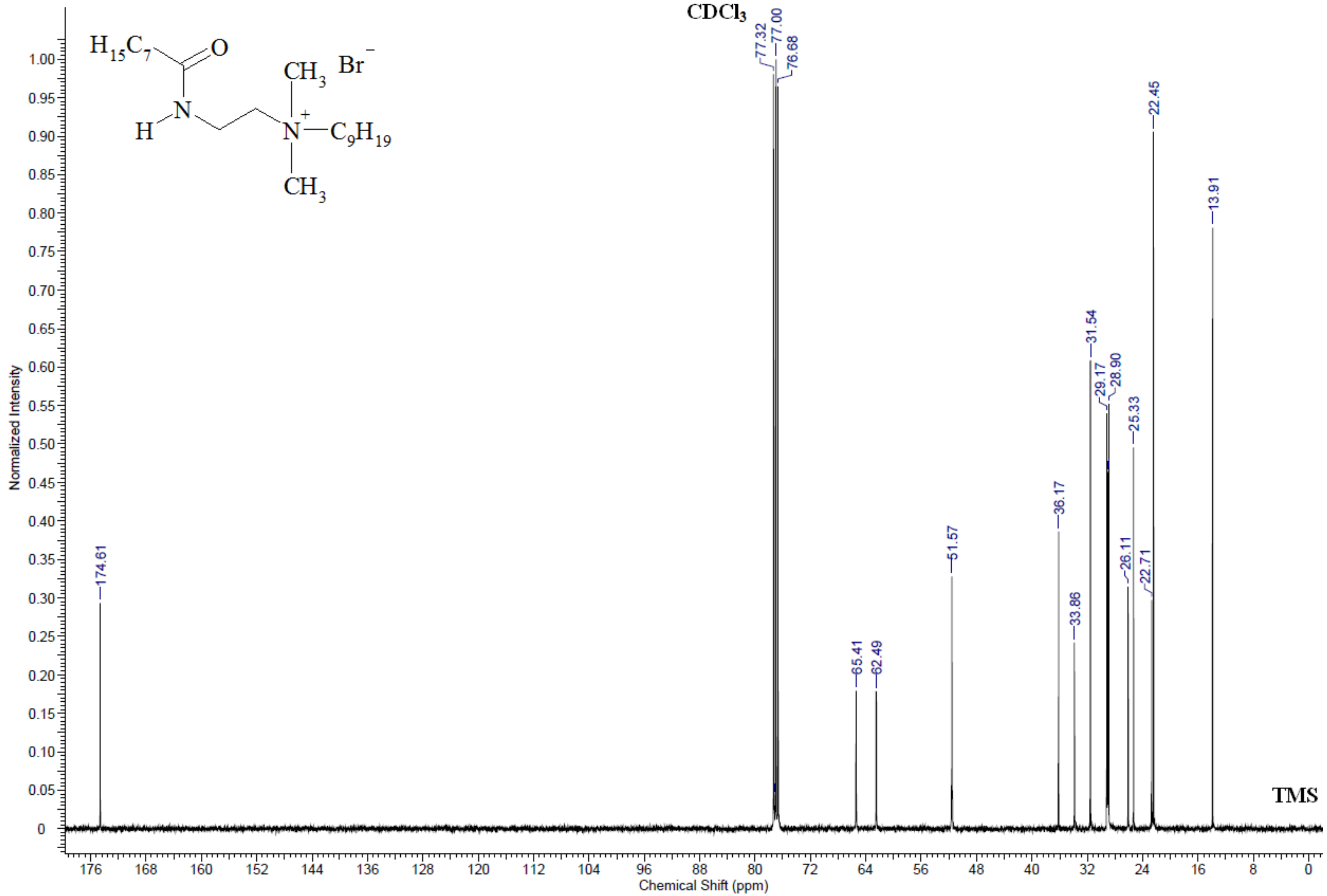

Fig. S8.^{13}C NMR spectrum of dimethylnonyl-*N*-[(2-octanamido)ethyl]ammonium bromide (**AC9**).

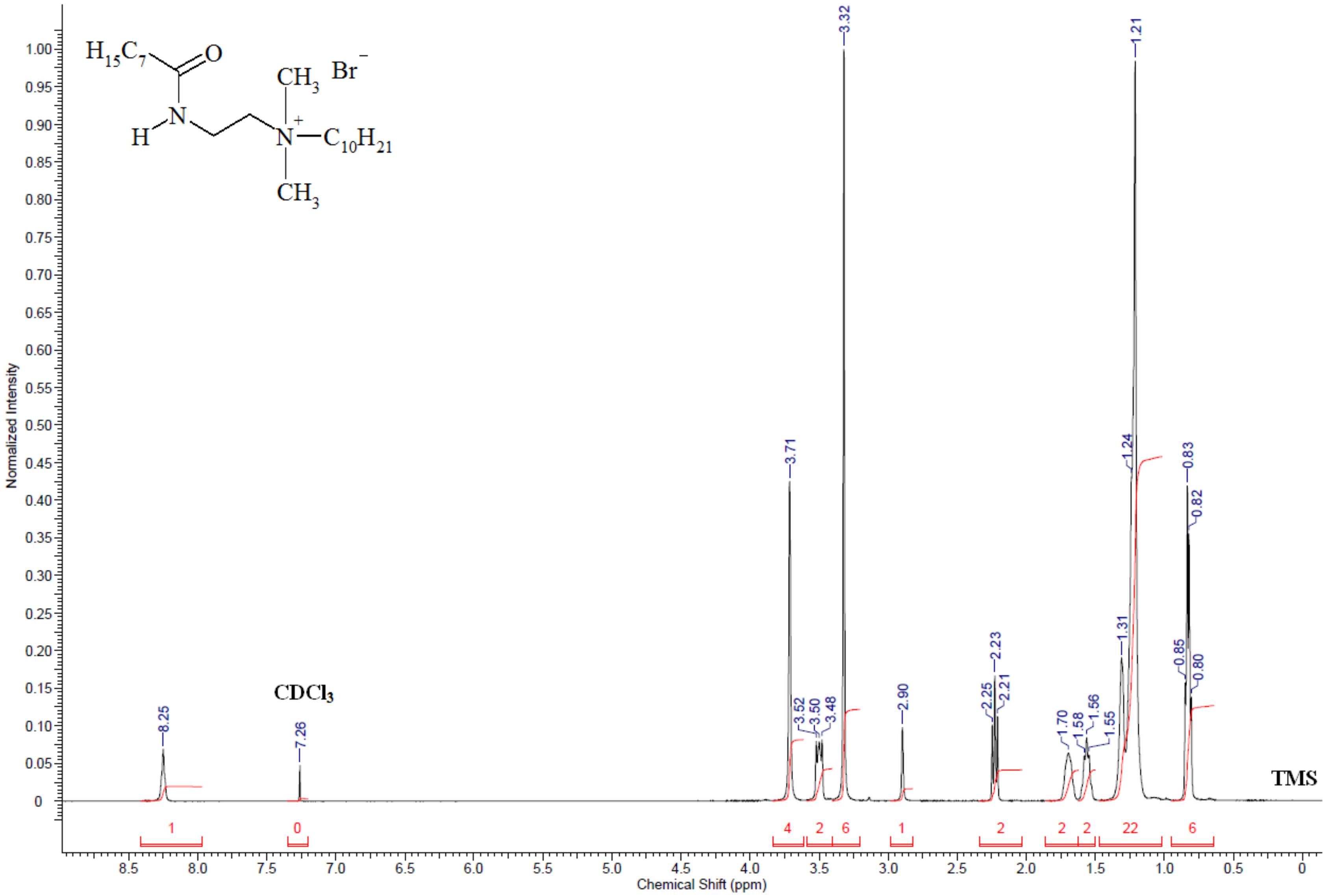

Fig. S9. ^{1}H NMR spectrum of decyldimethyl-*N*-[(2-octanamido)ethyl]ammonium bromide (**AC10**).

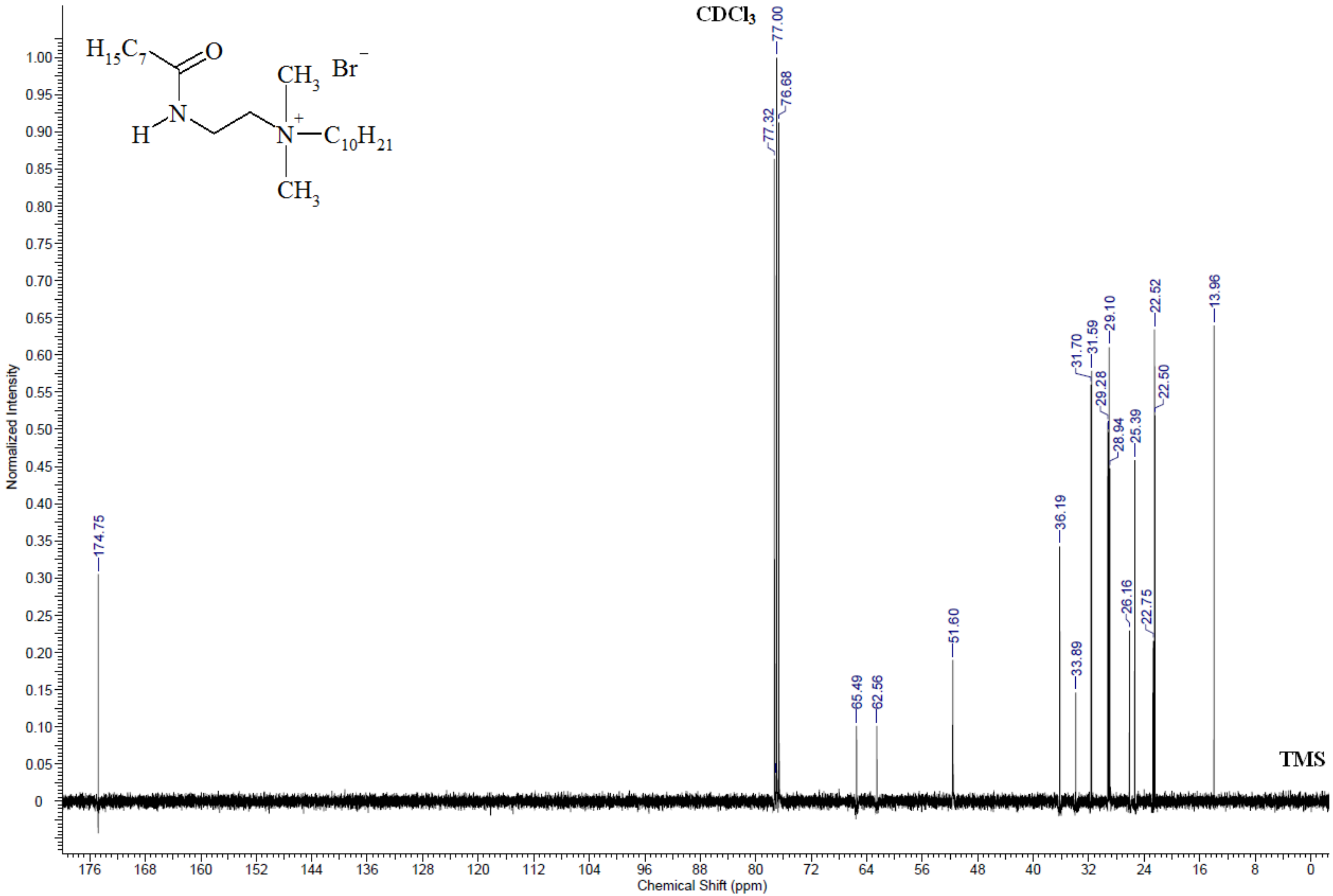

Fig. S10.^{13}C NMR spectrum of decyldimethyl-*N*-[(2-octanamido)ethyl]ammonium bromide (**AC10**).

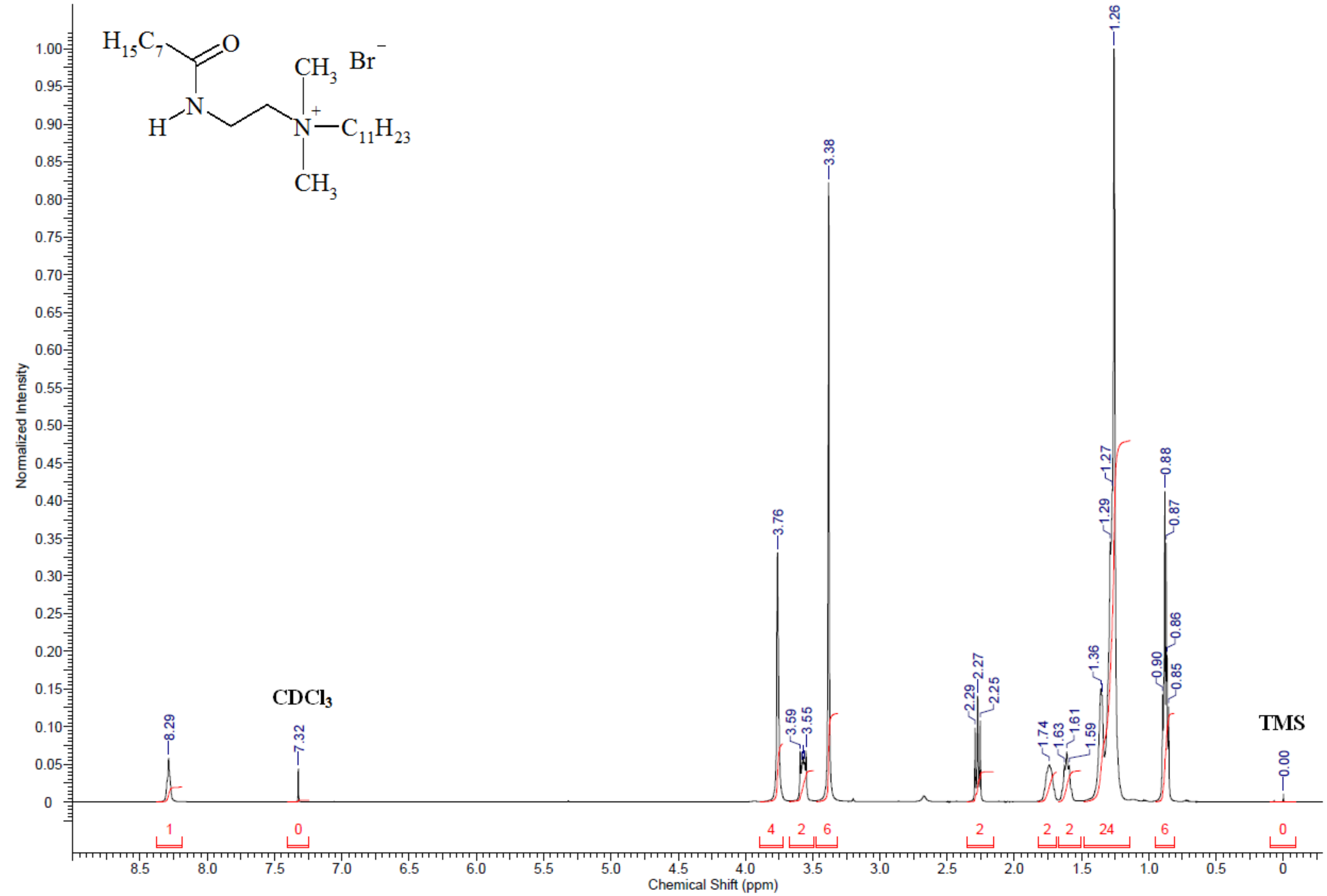

Fig. S11. ^{1}H NMR spectrum of dimethyl-*N*-[(2-octanamido)ethyl]undecylammonium bromide (**AC11**).

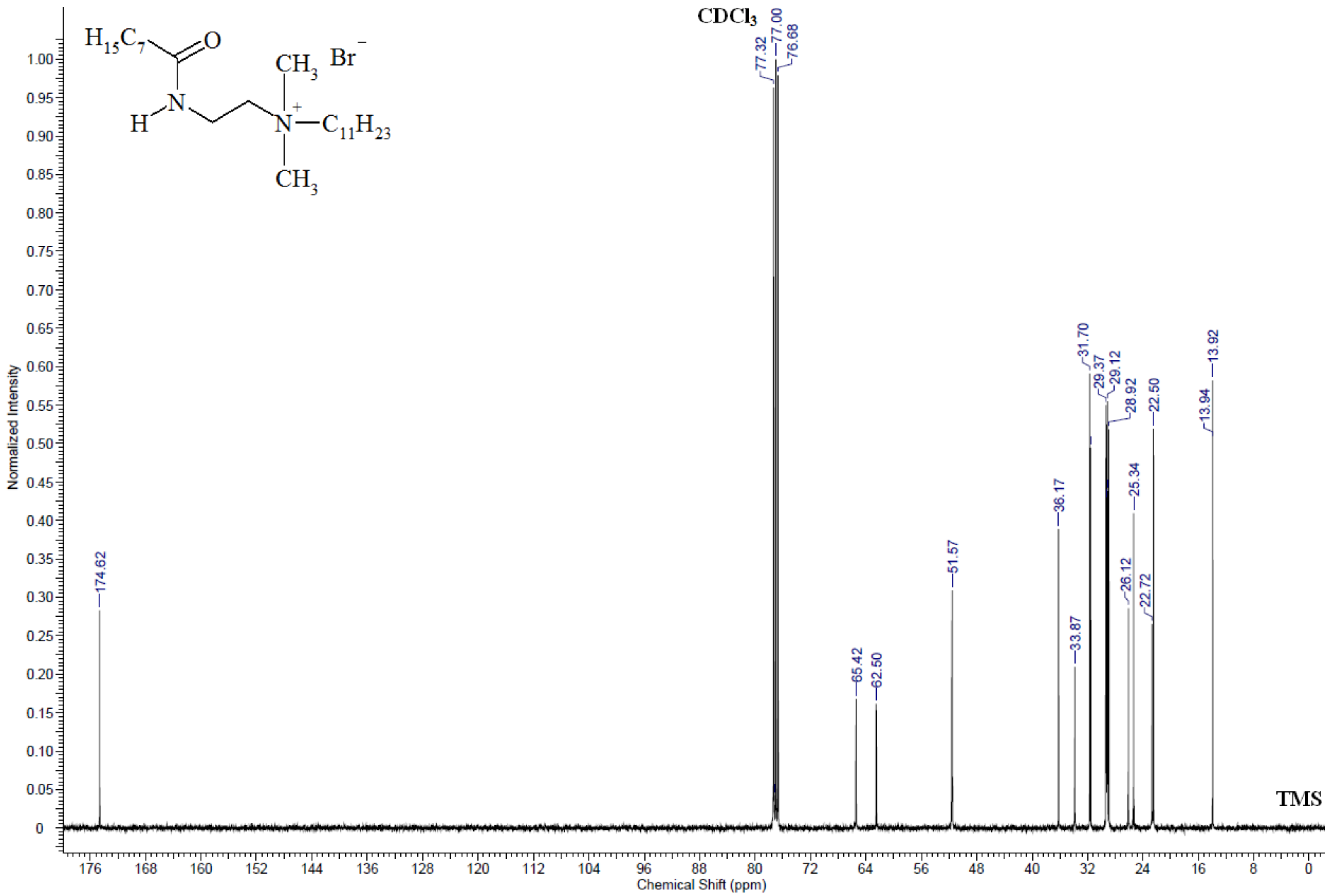

Fig. S12. ^{13}C NMR spectrum of dimethyl-*N*-[(2-octanamido)ethyl]undecylammonium bromide (**AC11**).

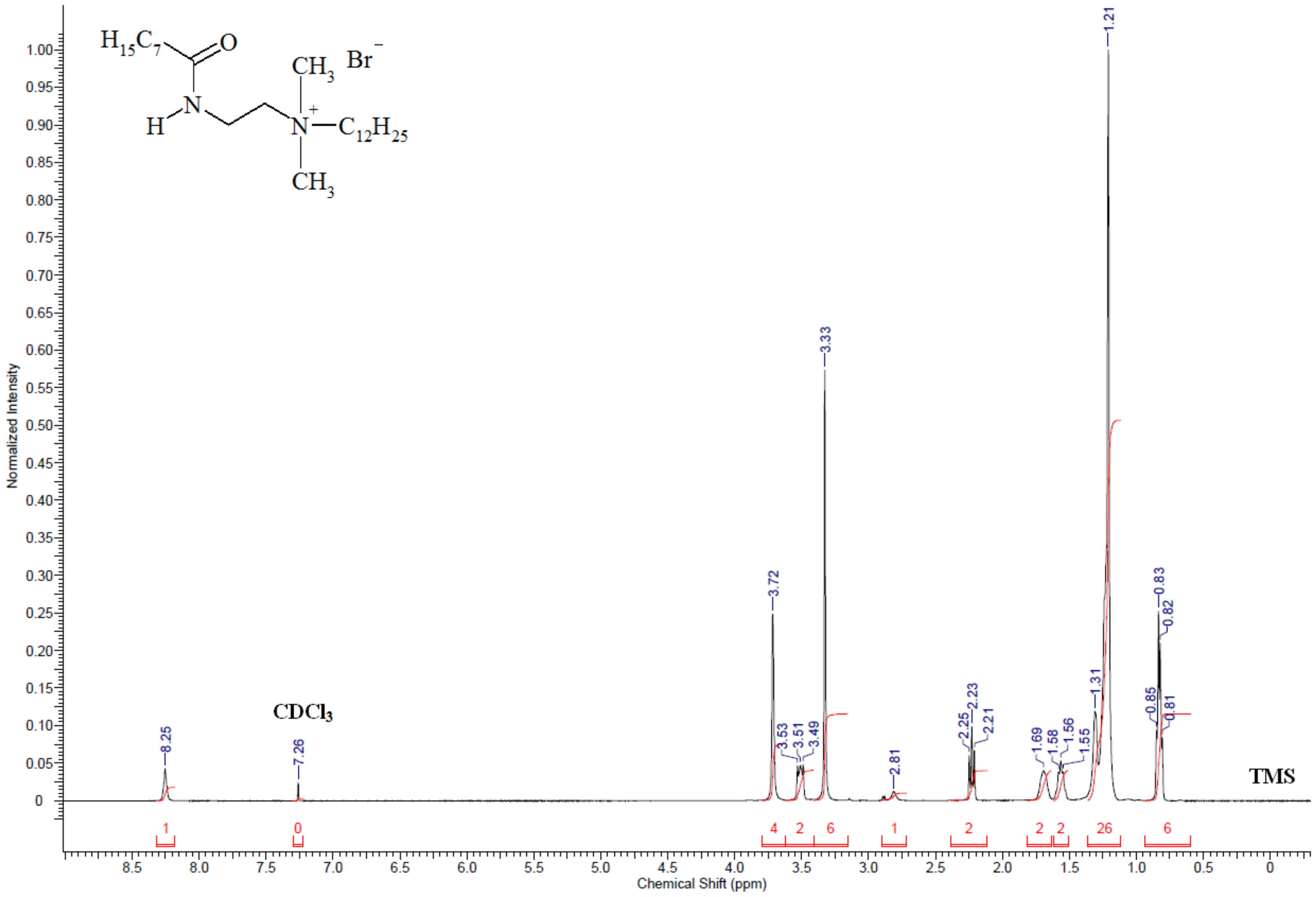

Fig. S13. ^{1}H NMR spectrum of dodecyldimethyl-*N*-[(2-octanamido)ethyl]ammonium bromide (**AC12**).

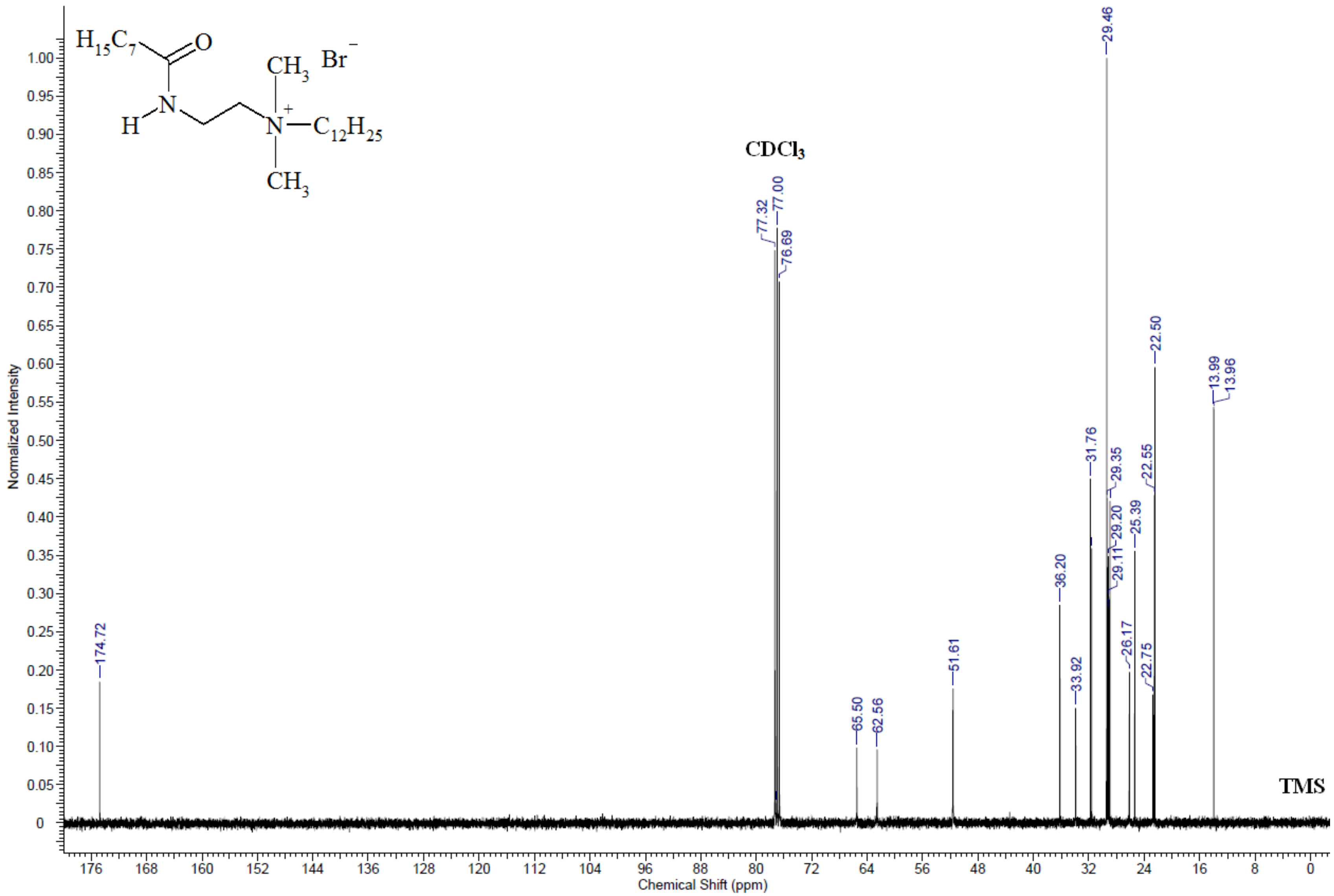

Fig. S14. ^{13}C NMR spectrum of dodecyldimethyl-*N*-[(2-octanamido)ethyl]ammonium bromide (**AC12**).

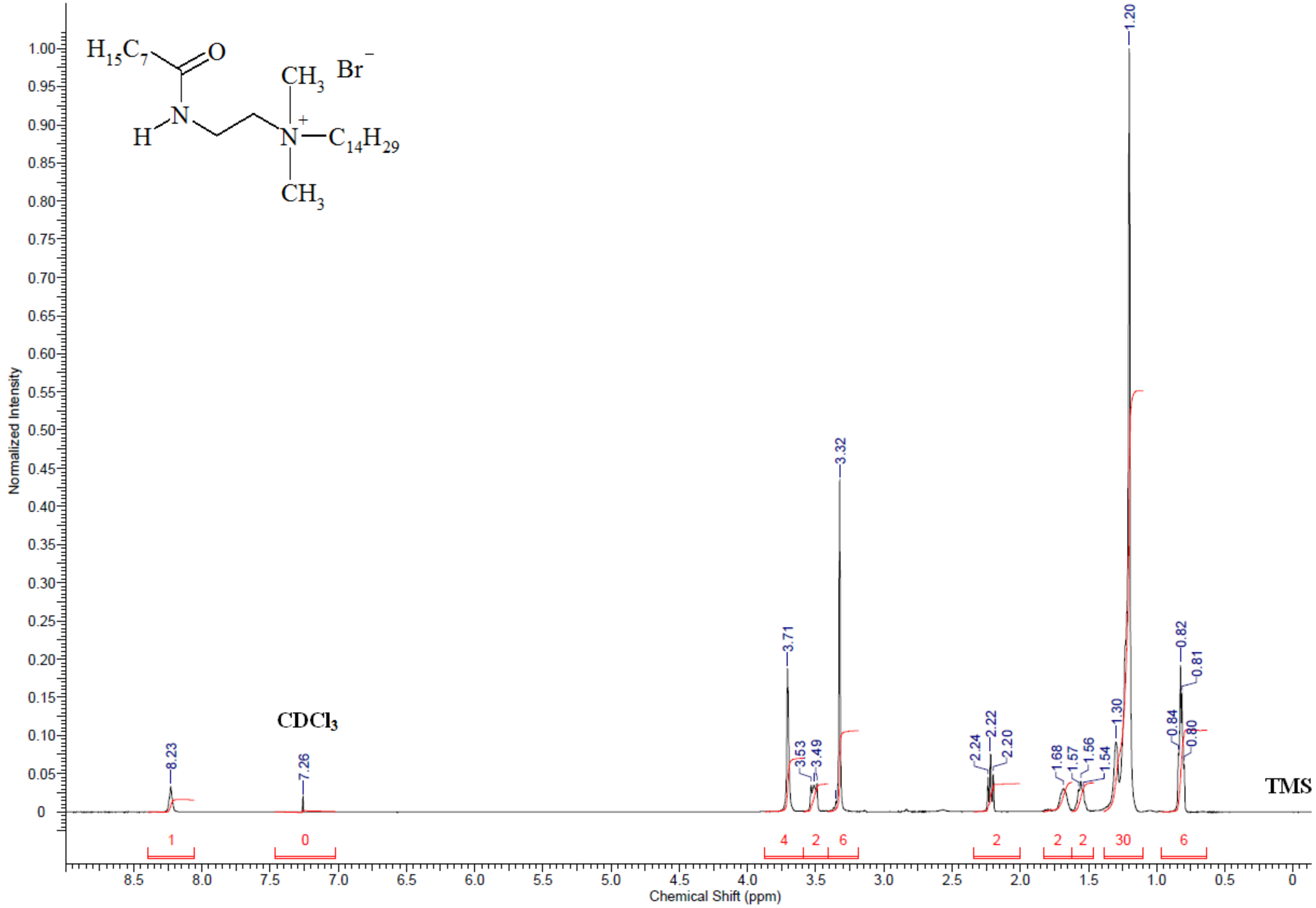

Fig. S15. ^{1}H NMR spectrum of dimethyl-*N*-[(2-octanamido)ethyl]tetradecylammonium bromide (**AC14**).

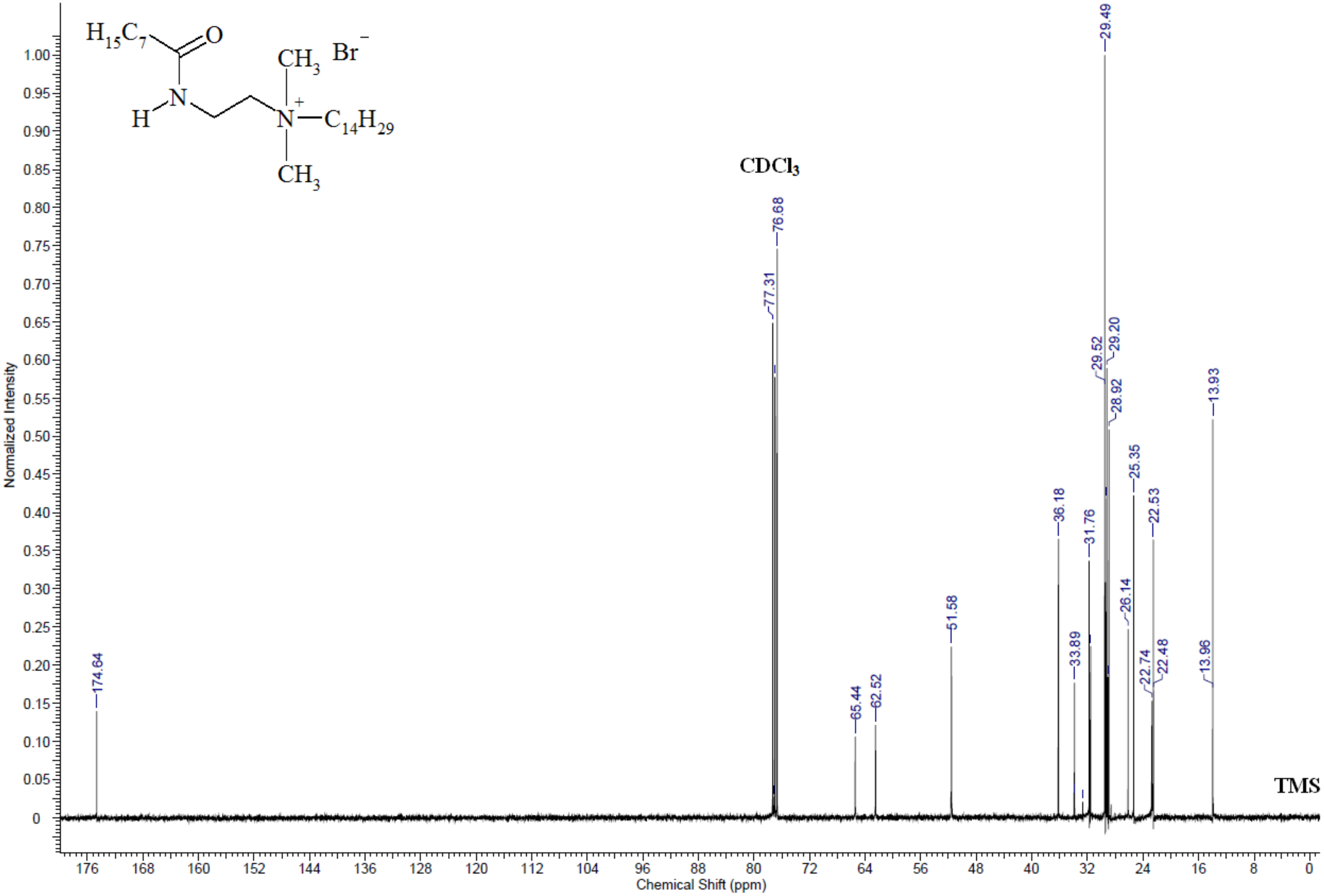

Fig. S16. ^{13}C NMR spectrum of dimethyl-*N*-[(2-octanamido)ethyl]tetradecylammonium bromide (**AC14**).

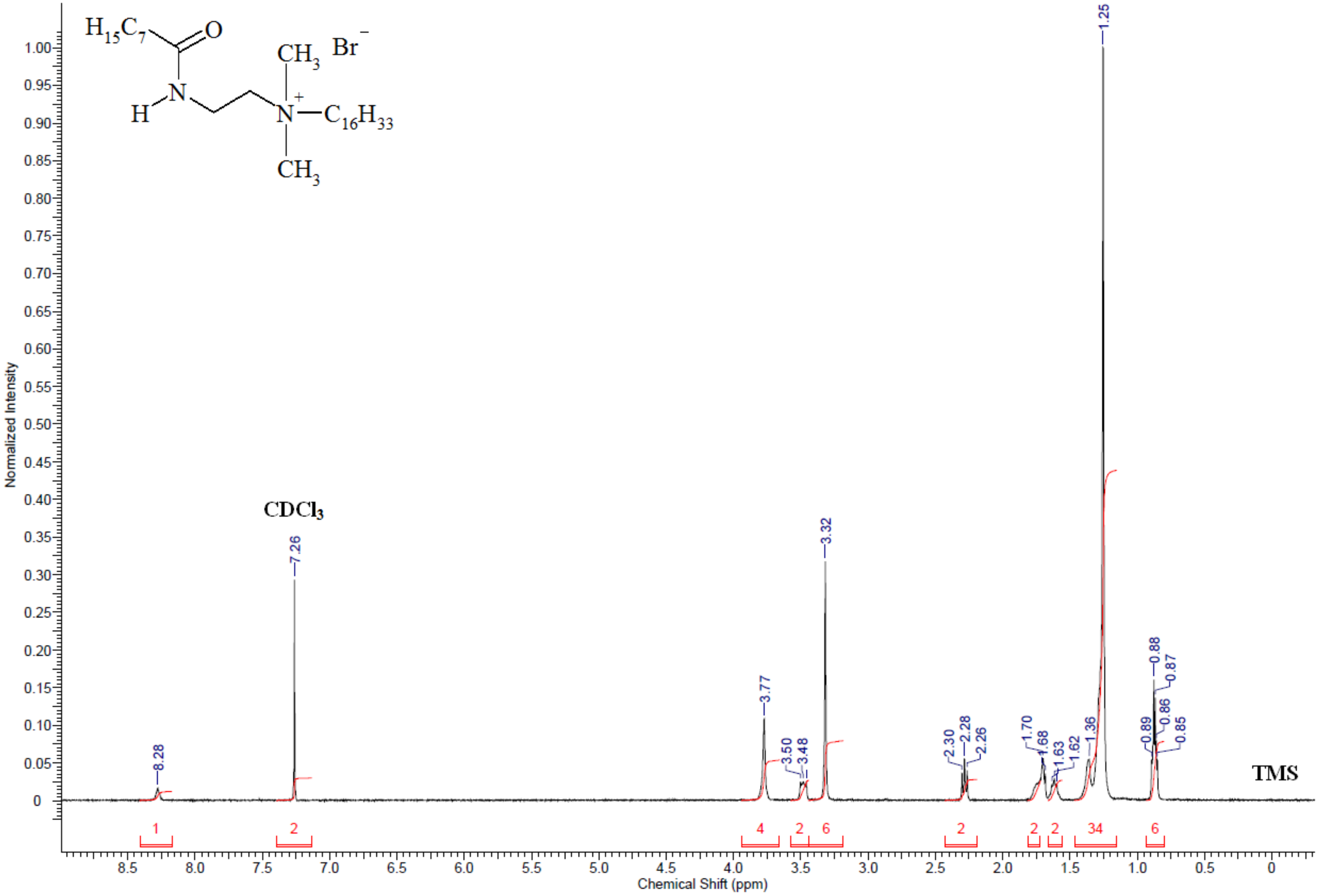

Fig. S17. ^{1}H NMR spectrum of hexadecyldimethyl-*N*-[(2-octanamido)ethyl]ammonium bromide (**AC16**).

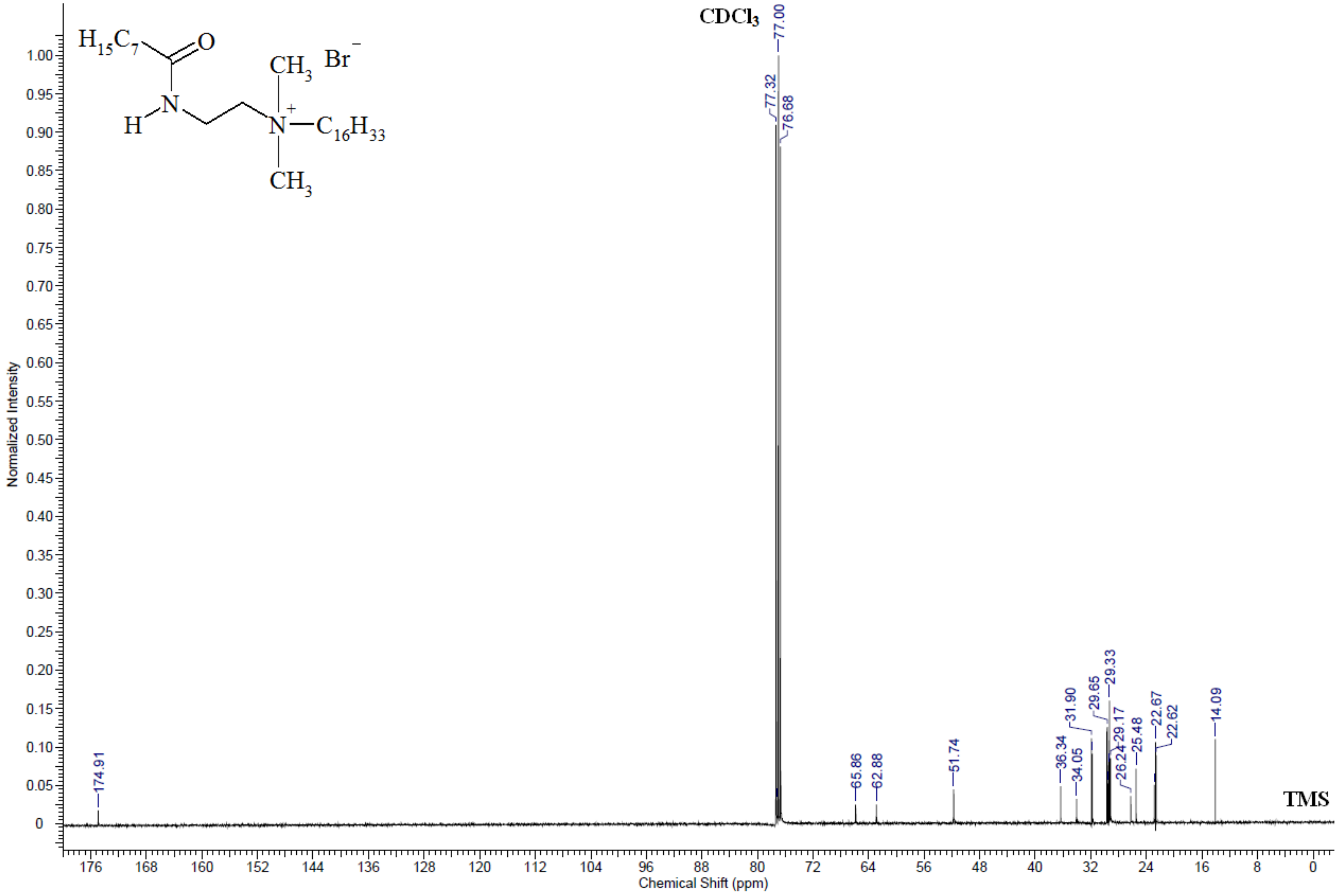

Fig. S18. ^{13}C NMR spectrum of hexadecyldimethyl-*N*-[(2-octanamido)ethyl]ammonium bromide (**AC16**).

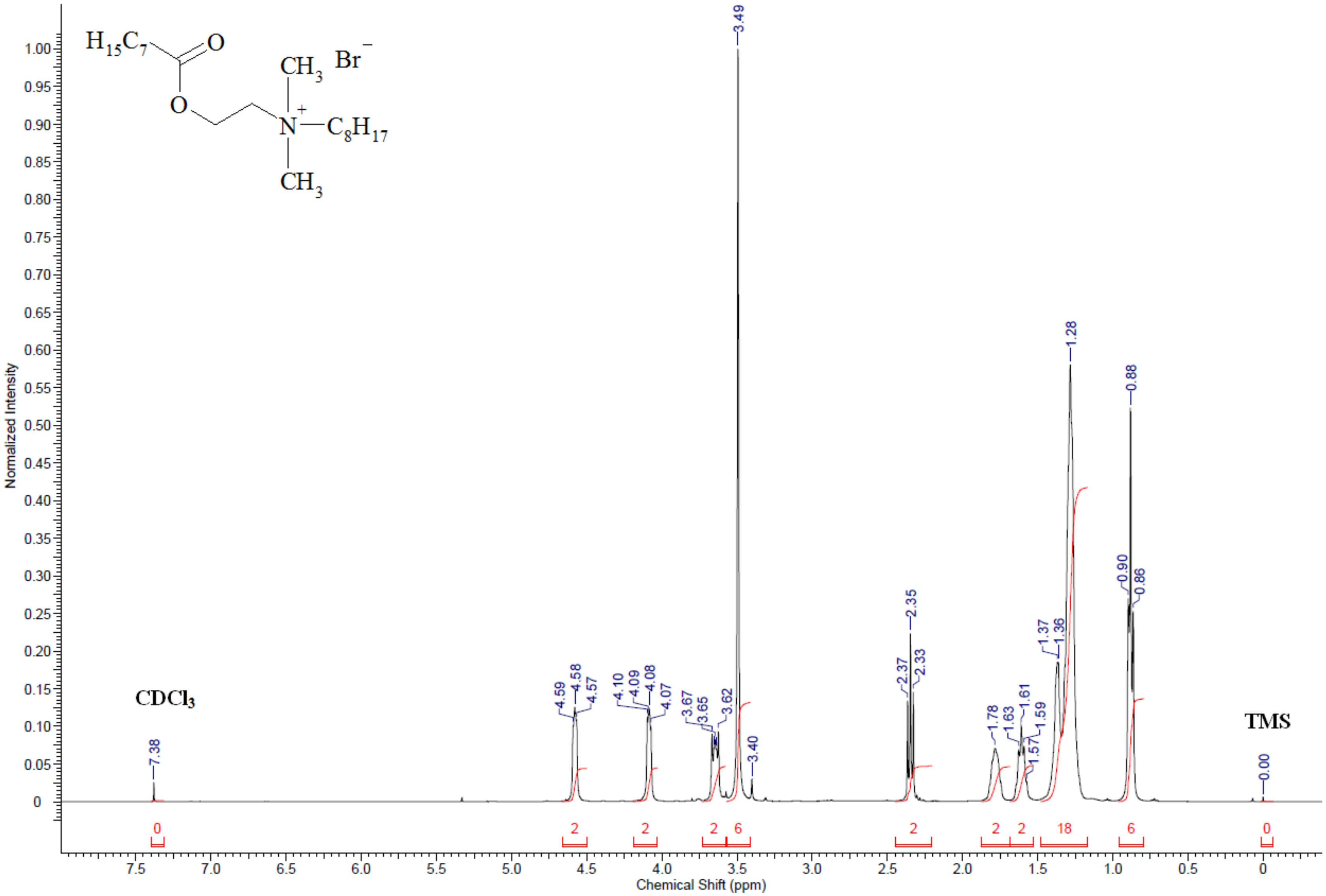

Fig. S19. ^{1}H NMR spectrum of dimethyl-2-octanoyloxyethyloctylammonium bromide (**EC8**).

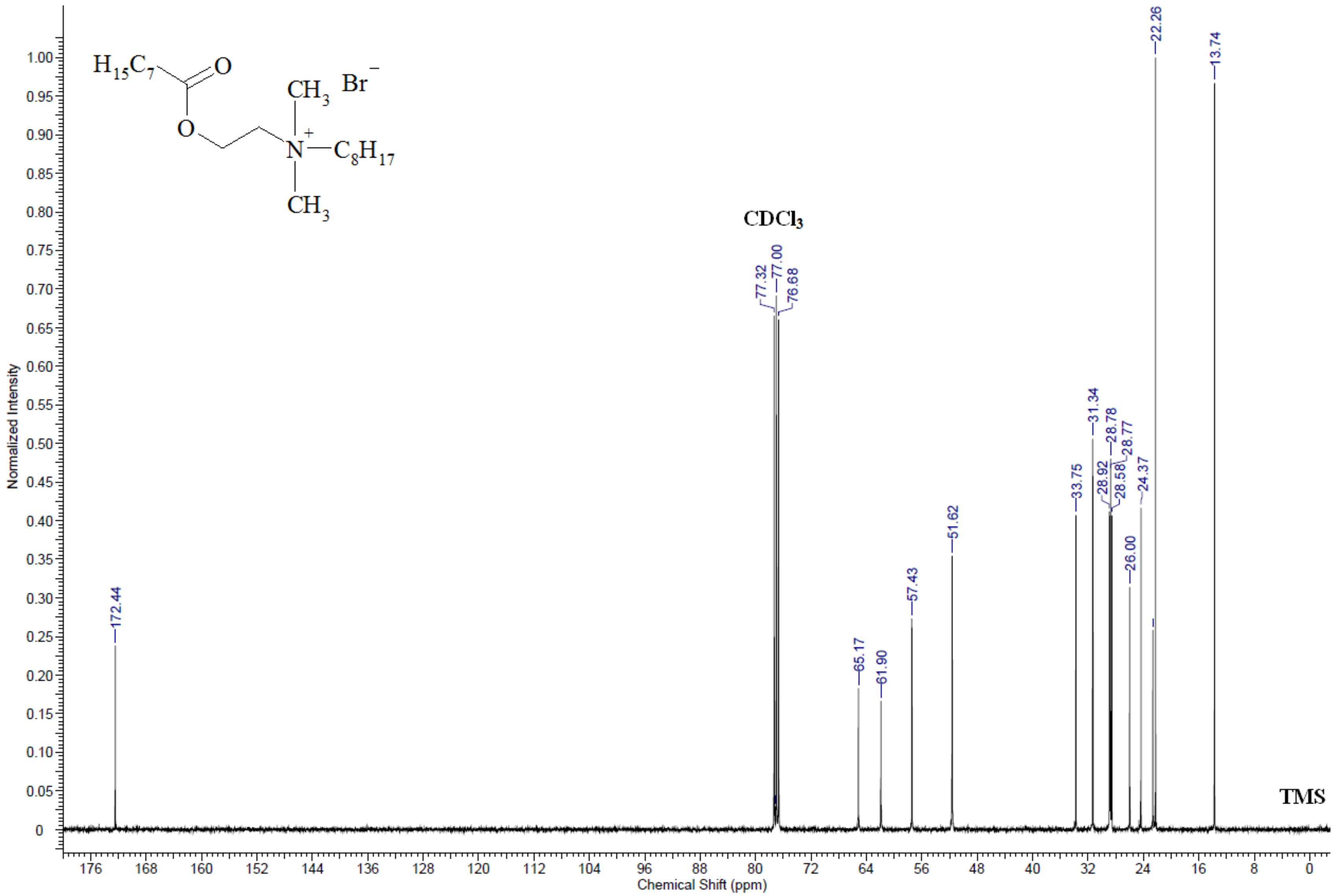

Fig. S20. ^{13}C NMR spectrum of dimethyl-2-octanoyloxyethyloctylammonium bromide (**EC8**).

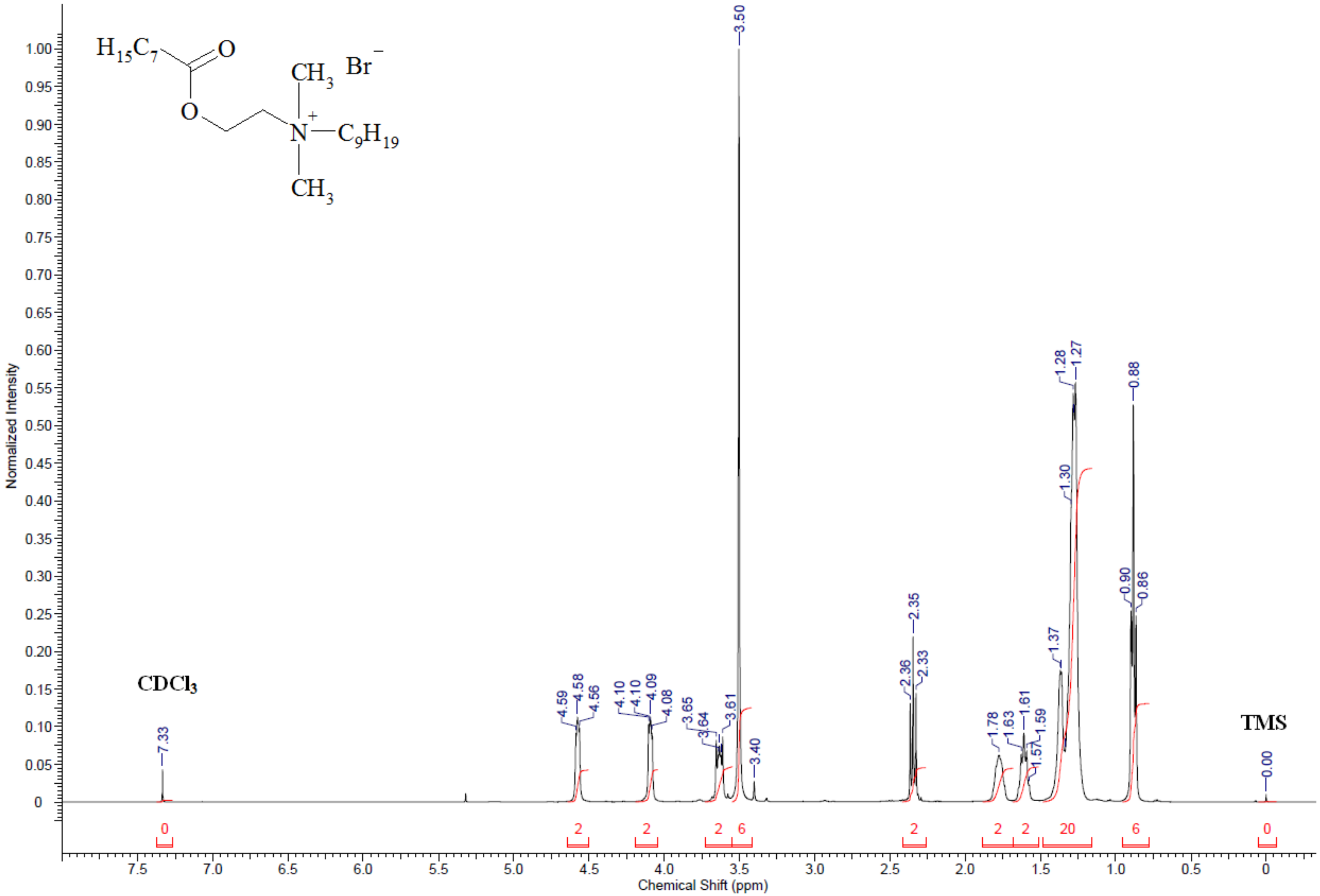

Fig. S21. ^{1}H NMR spectrum of dimethylnonyl-2-octanoyloxyethylammonium bromide (**EC9**).

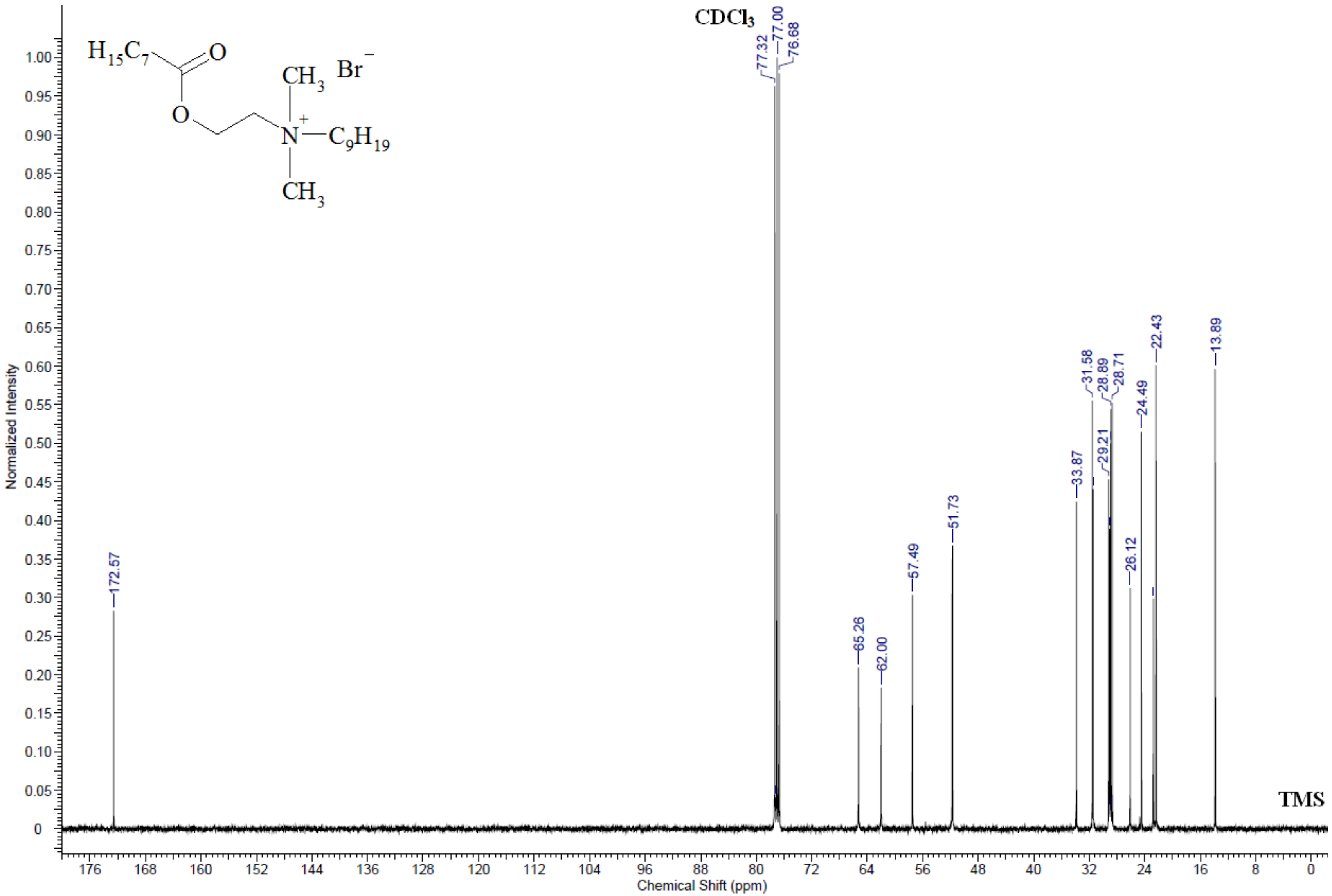

Fig. S22. ^{13}C NMR spectrum of dimethylnonyl-2-octanoyloxyethylammonium bromide (**EC9**).

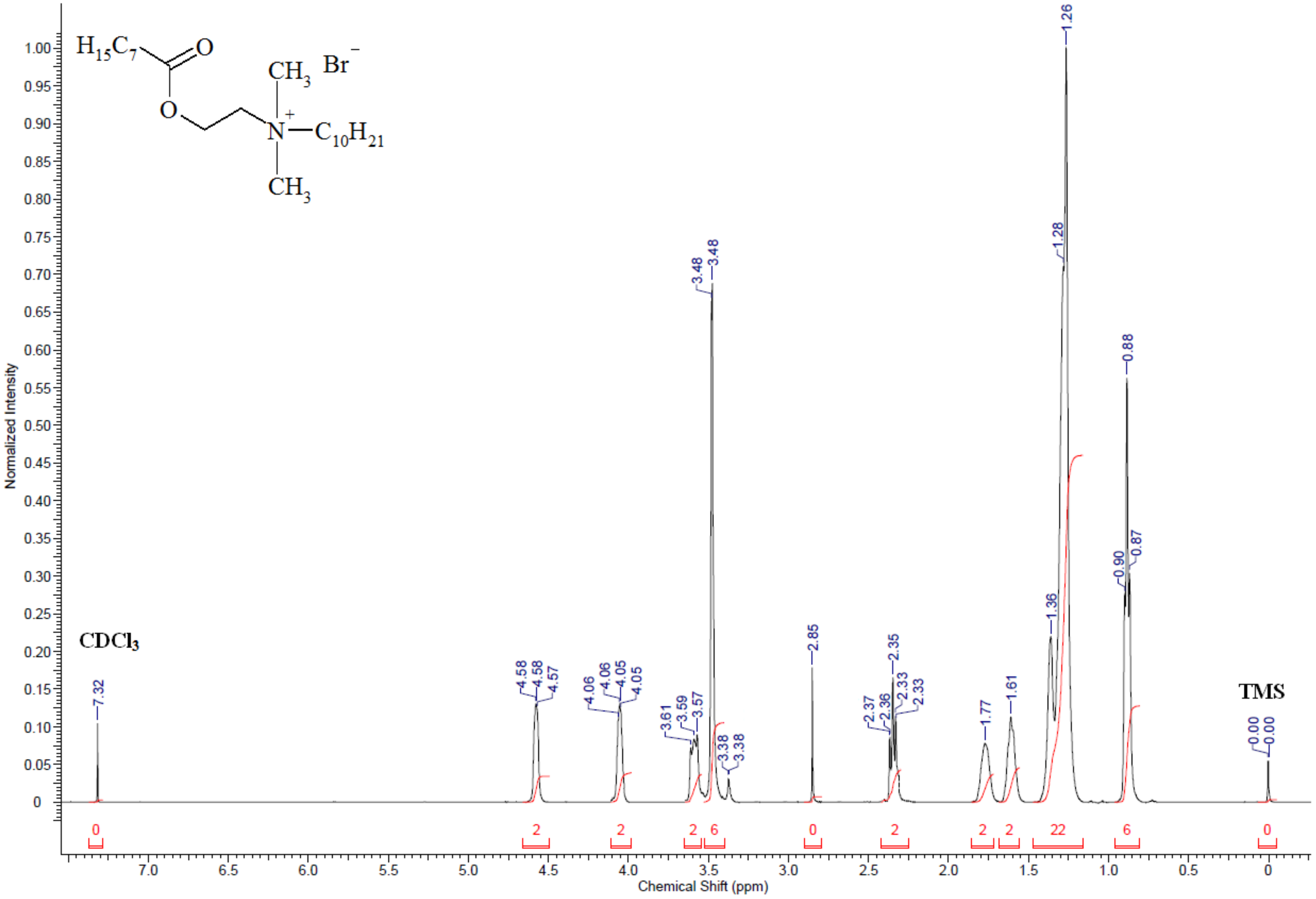

Fig. S23. ^{1}H NMR spectrum of decyldimethyl-2-octanoyloxyethylammonium bromide (**EC10**).

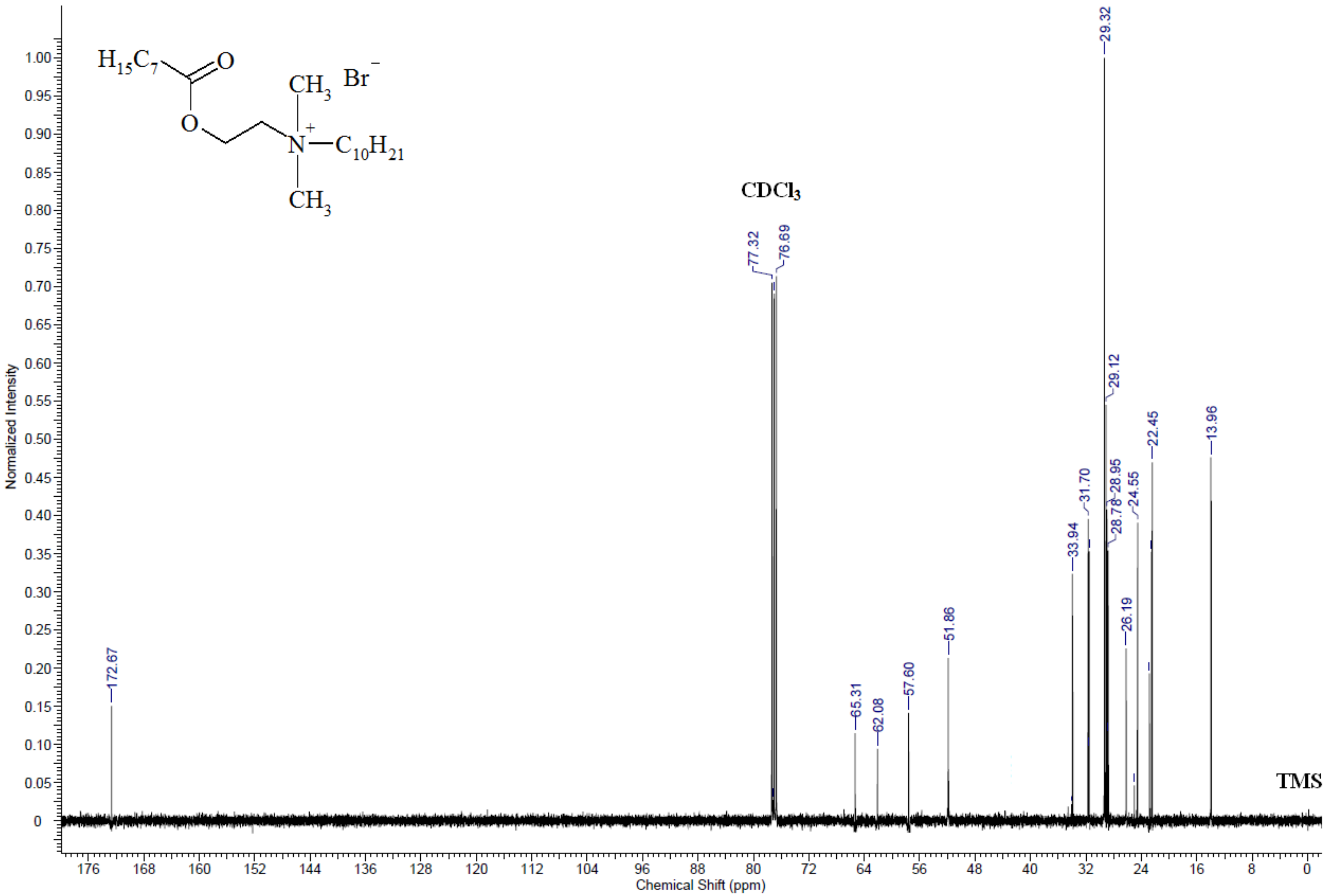

Fig. S24. ^{13}C NMR spectrum of decyldimethyl-2-octanoyloxyethylammonium bromide (**EC10**).

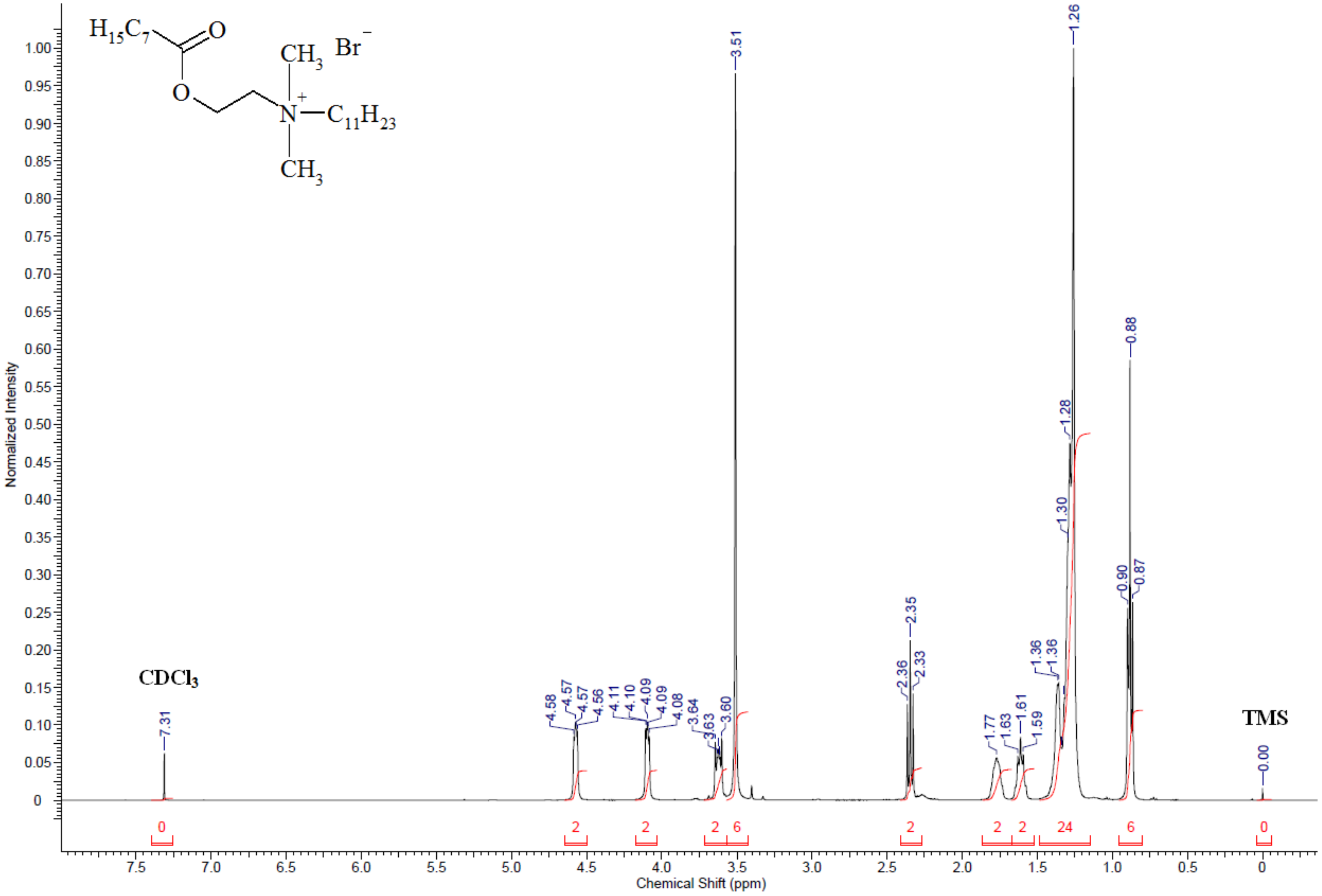

Fig. S25. ^{1}H NMR spectrum of dimethyl-2-octanoyloxyethylundecylammonium bromide (**EC11**).

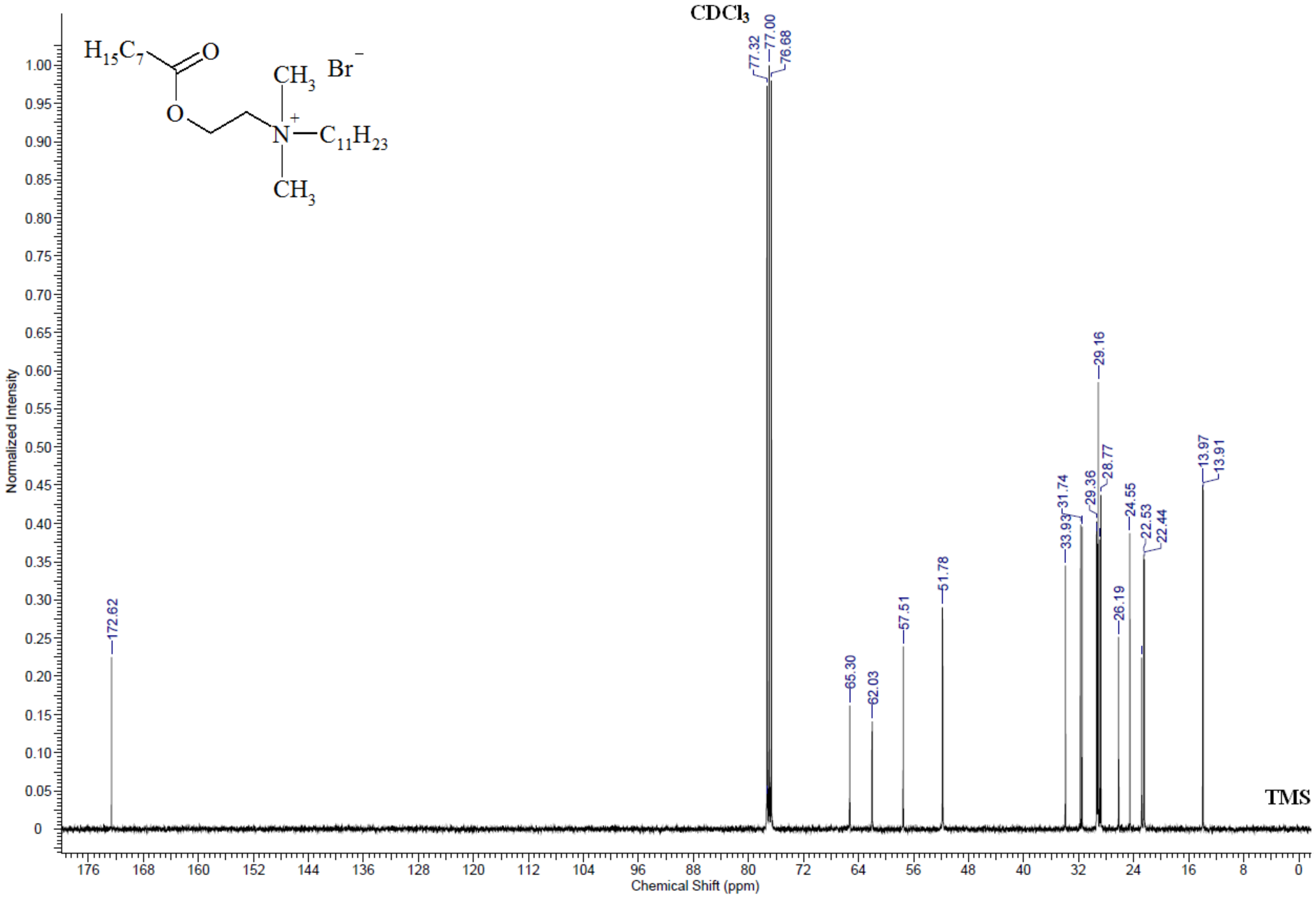

Fig. S26. ^{13}C NMR spectrum of dimethyl-2-octanoyloxyethylundecylammonium bromide (**EC11**).

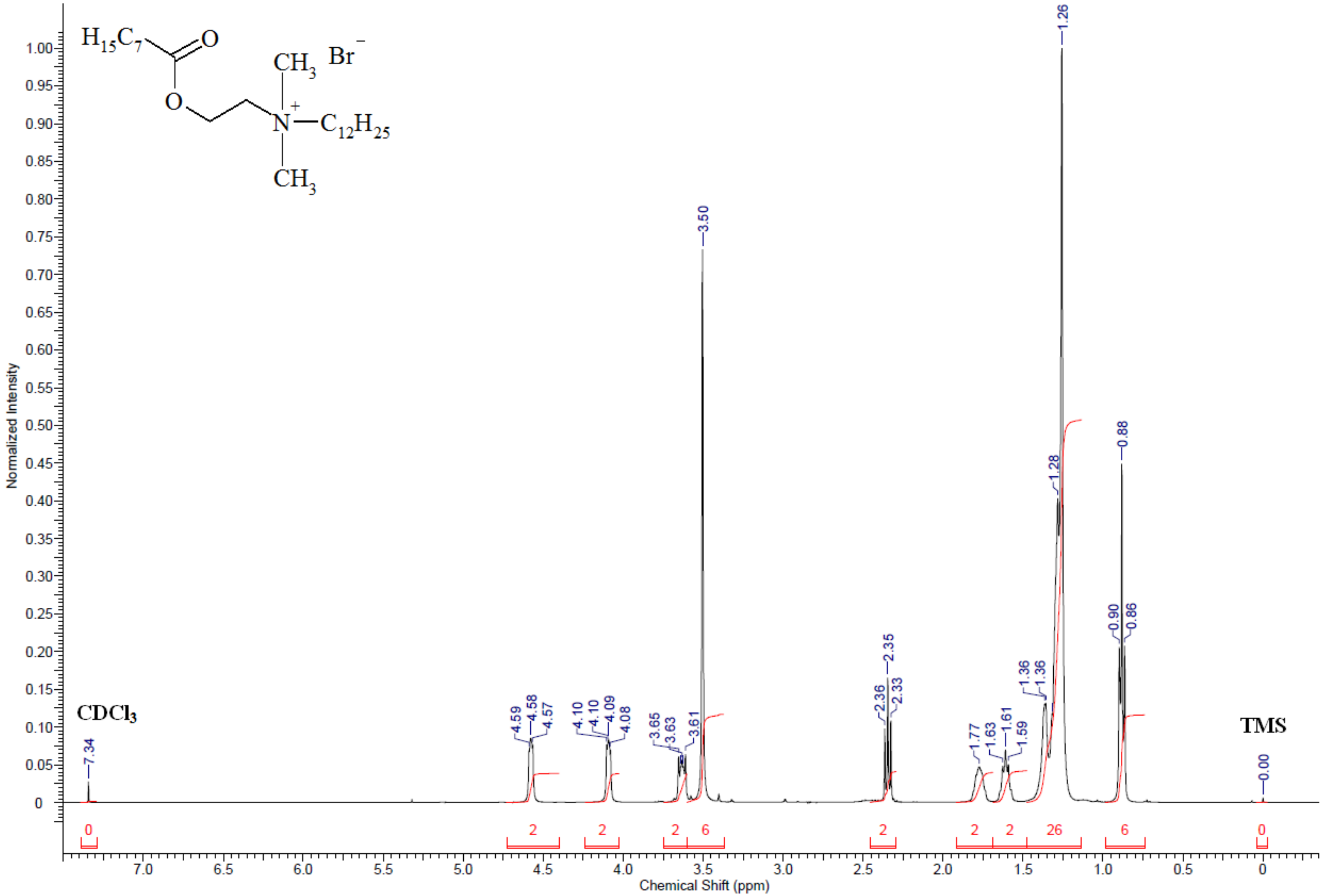

Fig. S27. ^{1}H NMR spectrum of dodecyldimethyl-2-octanoyloxyethylammonium bromide (**EC12**).

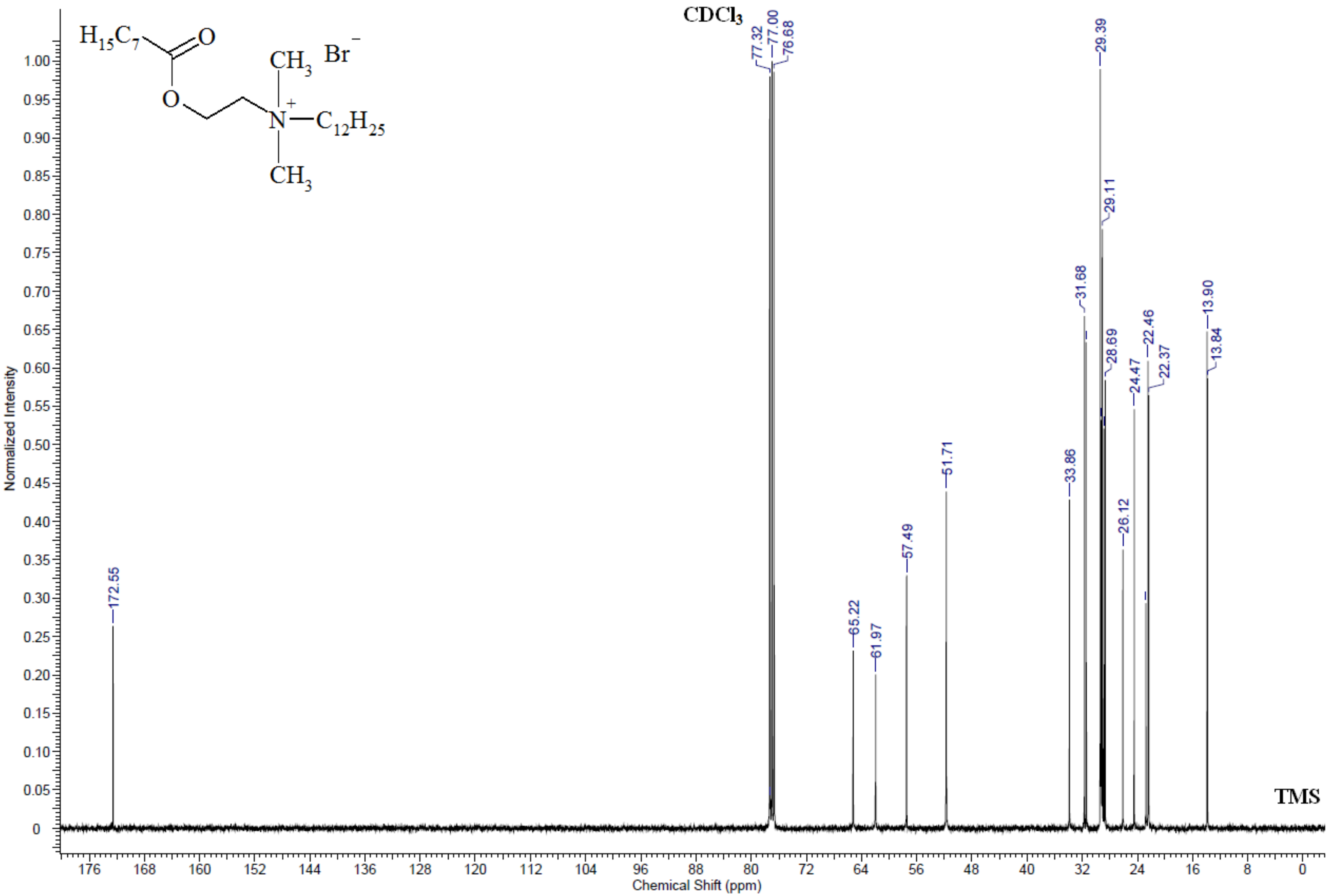

Fig. S28. ^{13}C NMR spectrum of dodecyldimethyl-2-octanoyloxyethylammonium bromide (**EC12**).

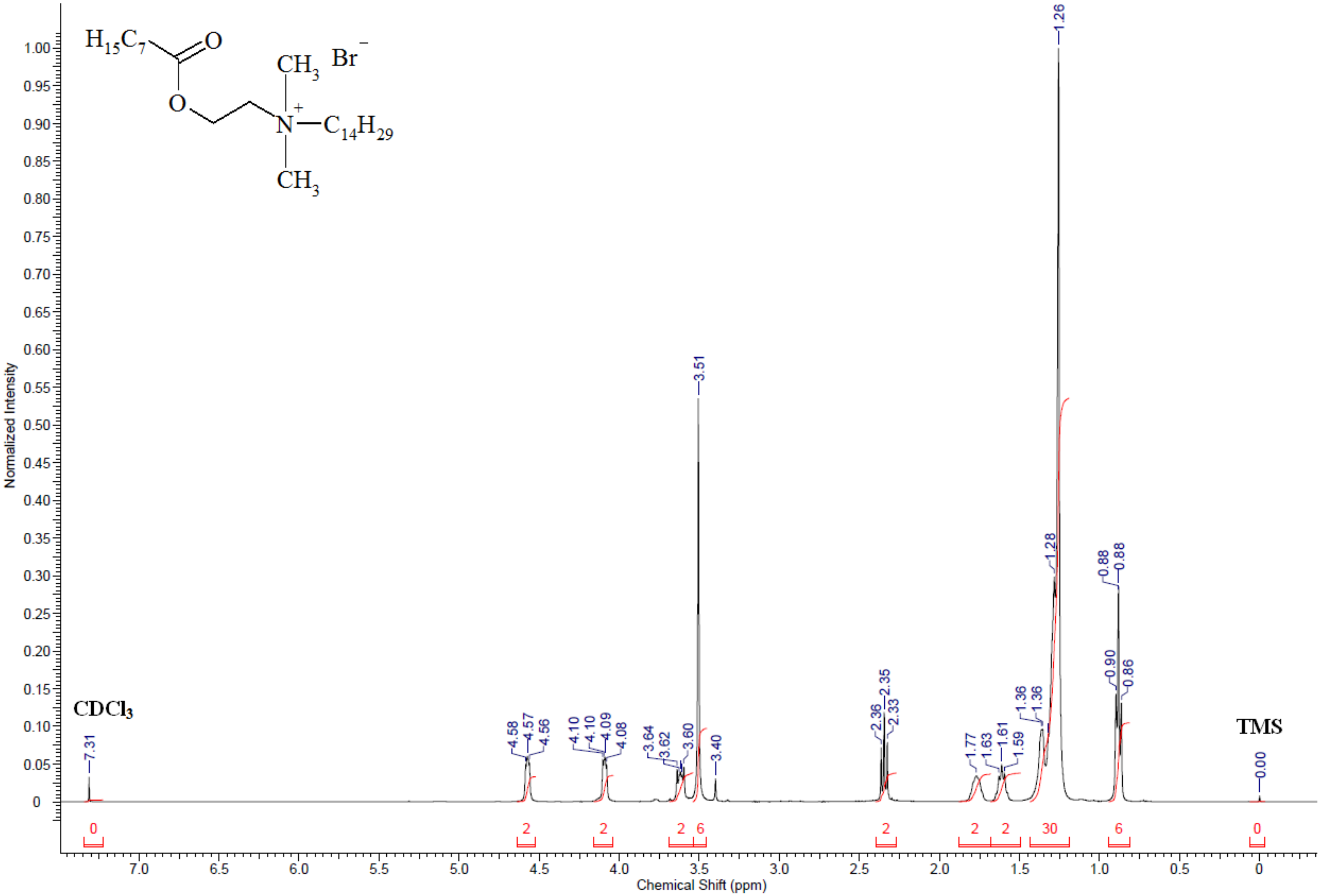

Fig. S29. ^{1}H NMR spectrum of dimethyl-2-octanoyloxyethyltetradecylammonium bromide (**EC14**).

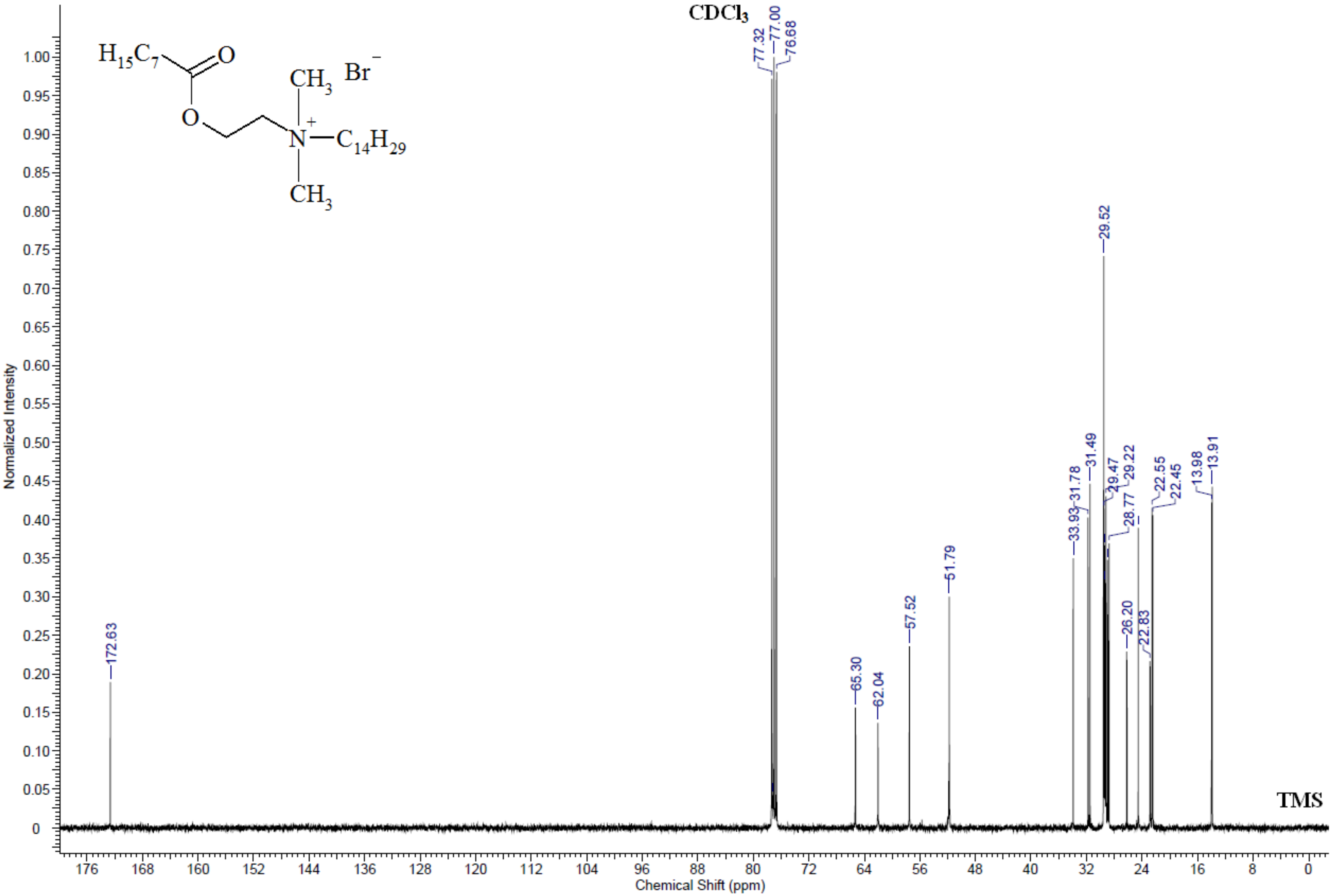

Fig. S30. ^{13}C NMR spectrum of dimethyl-2-octanoyloxyethyltetradecylammonium bromide (**EC14**).

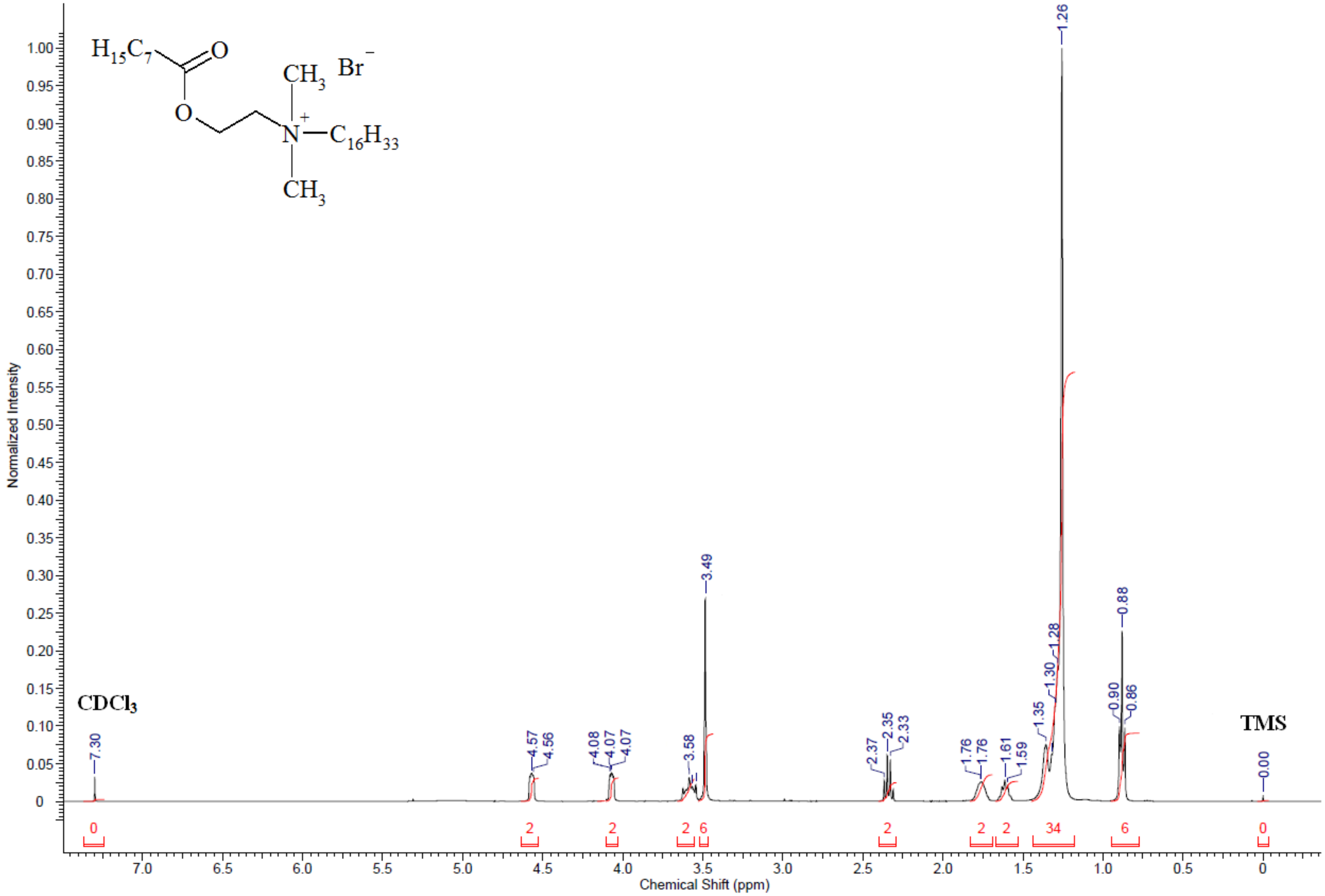

Fig. S31. ^{1}H NMR spectrum of hexadecyldimethyl-2-octanoyloxyethylammonium bromide (**EC16**).

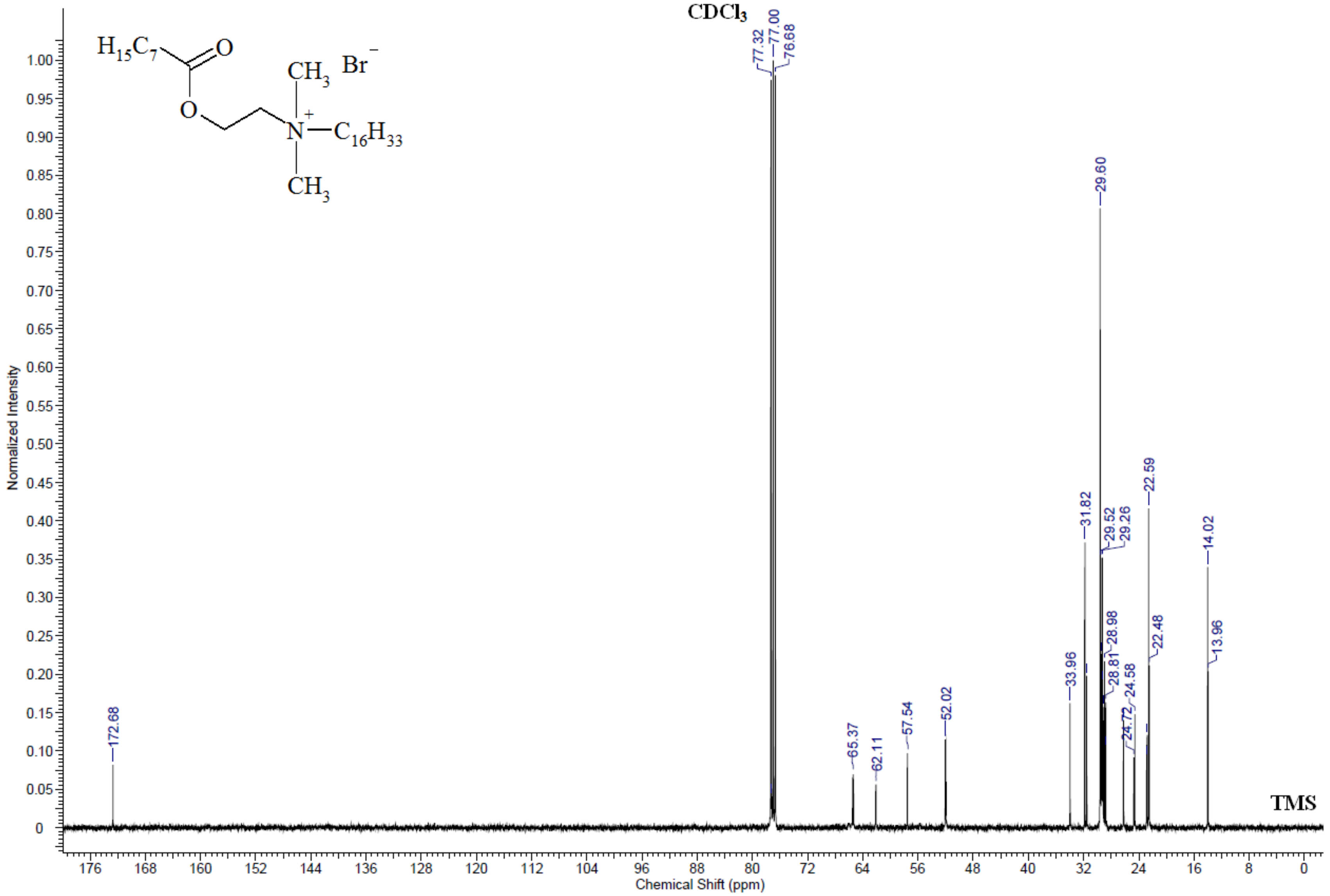

Fig. S32. ^{13}C NMR spectrum of hexadecyldimethyl-2-octanoyloxyethylammonium bromide (**EC16**).

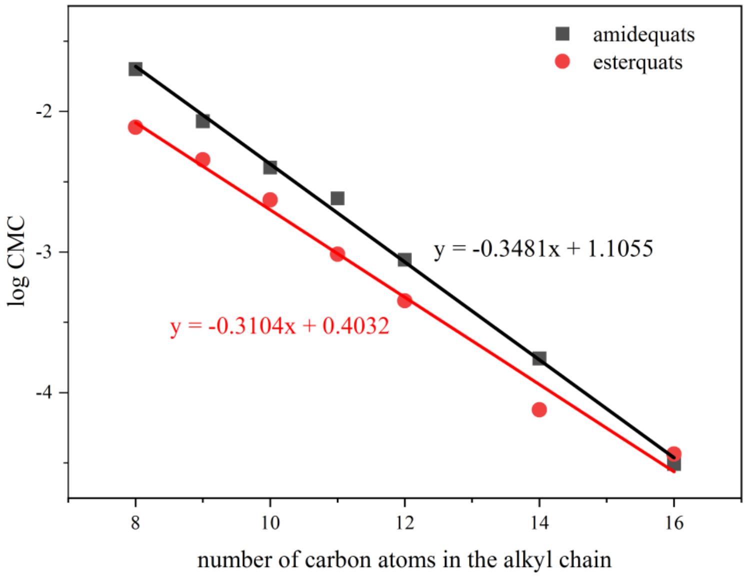

Fig. S33. Effect of the elongation of alkyl chain on the CMC of amidequats and esterquats.
